# Polygenic scores for longevity in relation to brain MRI measures in a cohort of 70-year-olds

**DOI:** 10.1038/s41514-026-00546-8

**Published:** 2026-10-15

**Authors:** Nazib M Seidu, Olof Lindberg, Silke Kern, Lina Rydén, Lars-Olof Wahlund, Margda Waern, Rosaleena Mohanty, Nira Cedres, Henrik Zetterberg, Kaj Blennow, Hanna Falk Erhag, Anna Zettergren, Eric Westman, Henne Holstege, Ingmar Skoog

**Affiliations:** 1 Neuropsychiatric Epidemiology (EPINEP), Centre for Ageing and Health (AgeCap), Institute of Neuroscience and Physiology, Sahlgrenska Academy, University of Gothenburg, Gothenburg, Sweden; 2 Departments of Radiology and Clinical Neurosciences, Hotchkiss Brain Institute, University of Calgary, Calgary, AB Canada; 3 Department of Neurobiology, Care Sciences and Society, Center for Alzheimer Research, Division of Clinical Geriatrics, Karolinska Institutet, Stockholm, Sweden; 4 Department of Neuropsychiatry, Sahlgrenska University Hospital, Gothenburg, Region Västra Götaland Sweden; 5 Department of Neuropsychiatry, Sahlgrenska University Hospital, Gothenburg, Sweden; 6 Region Västra Götaland, Psychosis Clinic, Gothenburg, Sweden; 7 UW Department of Medicine, School of Medicine and Public Health, Madison, WI USA; 8 Department of Psychology, Faculty of Health Sciences, University Fernando Pessoa-Canarias, Las Palmas, Spain; 9 Department of Psychiatry and Neurochemistry, Institute of Neuroscience and Physiology, Sahlgrenska Academy, University of Gothenburg, Gothenburg, Sweden; 10 Clinical Neurochemistry Laboratory, Sahlgrenska University Hospital, Region Västra Götaland, Gothenburg, Sweden; 11 UK Dementia Research Institute at UCL, London, UK; 12 Department of Neurodegenerative Disease, UCL Institute of Neurology, London, UK; 13 Hong Kong Center for Neurodegenerative Diseases, Hong Kong, China; 14 Department of psychology, SCI-LAB, Stockholm University, Stockholm, Sweden; 15 Research & Innovation Services at the University of Gothenburg Administration, Gothenburg, Sweden; 16 The Ageing Epidemiology Research Unit, School of Public Health, Imperial College London, London, UK; 17 Delft Bioinformatics Lab, Delft University of Technology, Delft, The Netherlands; 18 Section Genomics of Neurodegenerative Diseases and Aging, Department of Clinical Genetics, Vrije Universiteit Amsterdam, Amsterdam UMC, Amsterdam, The Netherlands; 19 Alzheimer Center Amsterdam, Department of Neurology, Amsterdam Neuroscience, Vrije Universiteit Amsterdam, Amsterdam UMC, Amsterdam, The Netherlands

**Keywords:** Diseases, Medical research, Neurology, Neuroscience

## Abstract

Population-based studies on the relation between polygenic longevity scores (PGLSs) and specific brain regions on magnetic resonance imaging (MRI) are few. We aimed to investigate this relationship in a population of 70-year-olds from Gothenburg, Sweden (*N* = 675). We constructed two PGLSs (one with and one without the *APOE* locus) using longevity GWAS summary statistics. MRI outcomes included total grey matter, white matter (WM), WM hyperintensities (WMHs), subcortical volumes, small vessel disease (SVD) score, cortical thicknesses, and fractional anisotropy (FA) fibre tracts. We adjusted for sex, age, principal components, and sex interactions, and corrected for multiple comparisons. We found no associations in the total sample. In males, both PGLSs were associated with smaller WMHs volume and lower SVD burden, and a thicker cortex of pars triangularis, pars opercularis, and insula. Our findings suggest a possible link between genetic propensity for longevity and lower cerebrovascular burden and certain cortical grey matter areas in males.

## Introduction

The contribution of heritability to becoming a septuagenarian has been estimated as 10–25%^1^, and to become a centenarian, as ~60%^1^. Genome-wide association studies (GWAS) identify several human longevity genes often involved in the modulation of age-related diseases or traits^1,2^. An individual genetic predisposition for longevity can be estimated through polygenic scores, which are a weighted sum of the number of risk or protective alleles an individual carries, calculated using GWAS summary statistics^2^. One way, which we use, to construct the polygenic longevity scores (PGLSs) is by using the polygenic risk score continuous shrinking method^3^. We used GWAS summary statistics from Deelen et al. (2019) that rigorously pull together individuals from several cohorts, where the longevity phenotypes are defined based on cohort life tables from census data for the appropriate country, sex, and birth cohort^2^. Using this approach, studies have reported that individuals with high PGLSs are more likely to have a healthy metabolic profile^4^, more often have long-lived parents^2,5^, higher education^6^ and better cardiovascular health^2,5^. Using another approach in a centenarian study, it was reported that a higher PGLS based on parental longevity was associated with reduced mortality across an age continuum^1^.

Ageing is associated with progressive disruption of brain structures and functioning. Among older adults, brain ageing on structural magnetic resonance imaging (MRI) is characterised by loss of cortical grey matter (GM)^7^, reduced subcortical volumes (*e.g.*, the hippocampus)^8^, an increase in white matter hyperintensities (WMHs)^9^, and alterations of microstructural white matter (WM) fibre tracts^10^. These structural brain changes are related to mortality^11,12^. In the same way, the *APOE* locus is strongly related to shorter survival^2^, and studies on healthy individuals have reported that *APOE* carriers often have smaller brain volumes^13,14^, thinner cortex^13,15,16^ and alterations of WM integrity^14^ compared to non-carriers, and females are more affected^13^. Furthermore, females live longer than males, and there are sex differences in how some cognitive abilities change throughout life^17^. Sex differences may thus influence changes in brain structures, maybe due to differences in hormone levels during life or social circumstances. Thus, it is important to test interaction with sex when examining associations between PGLSs and brain ageing processes^18^.

Apart from the *APOE* gene, GWAS has also identified other genes, *e.g.*, ASTN2, DPP4, MAST4 and MAPT, which are associated with brain MRI measures, including the hippocampus^19^, cortical thickness^20^, and diffusion MRI (dMRI) fibre tracts^20,21^. The finding that several genes are involved in brain ageing facilitates the use of polygenic risk scores (PRSs) to investigate age-related disease, including Alzheimer’s disease (AD) and structural brain changes. In this context, studies have found that higher scores in AD PRS are associated with lower hippocampal volume^22,23^, thinner brain cortex^24^, and microstructural alteration of dMRI tracts^23^. Despite this, few studies have examined PGLSs in relation to aspects of the ageing brain on MRI. Thus far, one study reported that higher PGLSs were associated with larger brain volume and lower levels of total WMHs volume measured by MRI^18^. This leads to the question; to what extent do individuals with high PGLSs also maintain brain structures representative of younger ages? Given the strong effect of the *APOE* locus on brain structures and risk of Alzheimer’s disease (AD) and mortality^2^, it is important to examine PGLSs with and without the *APOE* locus. We would expect that the non-*APOE* PGLS might have less effect on brain structures than the PGLS, including the *APOE*.

To further explore this association, we examined the association between our primary exposure, the PGLS that included the *APOE* locus (*APOE* PGLS) and our primary outcomes^1^; brain MRI measures supposed to reflect cerebrovascular changes (WMHs, small vessel disease (SVD) score, and mean fractional anisotropy (FA) in WM fibre tracts value), and^2^ brain MRI measures supposed to reflect neurodegeneration of relevance for ageing (total GM, WM, and subcortical volumes, and mean cortical thickness value) in a population-based sample of cognitively healthy 70-year-olds in Sweden. Analyses were performed in the total sample and stratified by sex if sex interactions were significant. We also performed analyses by examining the secondary exposure, the non-*APOE* PGLS^2^, in relation to the primary MRI outcomes.

In secondary analyses, we repeated the analyses by exploring the relation between the two PGLSs and the secondary MRI outcomes that included regional subcortical volumes, regional cortical thicknesses, and FA WM fibre tracts. In addition, we also did some sensitivity analyses between both PGLSs and our primary outcomes, including log-transformed WMHs volume, SVD score without WMHs, the raw SVD score on an ordinal scale(0-4), and adjusted both the vascular primary outcomes (total WMHs volumes and SVD score) models for additional vascular and lifestyle-related factors (years of education, smoking, hypertension, BMI, diabetes, and hypercholesterolaemia).

## Results

Among the 675 individuals with available MRI at the baseline examination (Table 1), 51.6% were females, 33.3% were *APOE ε4* carriers, the mean age was 70.8 years (SD = 0.33), the mean length of education was 13.4 years (SD = 4.1), the mean BMI was 25.8 (SD = 4.3), and the mean MMSE score was 29.1 (SD = 1.3). Characteristics by sex are given in Table 1. Males differed from females in MMSE score, diabetes, and atrial fibrillation (*P* < 0.04). On the other hand, there were no differences in age, length of education in years, BMI, *APOE ε4* carriership, current and lifetime smoking, hypercholesterolaemia, and hypertension. Compared to the primary MRI measures in females, males had larger total GM, WM, and subcortical volumes, larger ICV, smaller mean cortical thickness value, higher total WMHs volume, higher log total WMHs volume, more EPVS in the basal ganglia, and higher total SVD burden (P < 0.03). In the supplementary materials sTable 2, we showed the number of SVD events by sex and both PGLSs. sFigure 1 and 2 show the graphical distribution for the total WMHs volumes, log total WMHs volume, total SVD score and SVD score without WMHs. sFigure 3 shows the distribution of the PGLSs in males and females, and there were no sex differences.

**Table 1:** Participants with both genetic and brain MRI data characteristics (*N* = 675)

Variables	Total sample (*N* = 675)	Males (*n* = 327)	Females (*n* = 348)	*P* value
**Demographics: mean (SD)**				
Age (in years)	70.8 (0.33)	70.8 (0.34)	70.8 (0.33)	0.6
Education (in years)	13.4 (4.1)	13.5 (4.22)	13.4 (4.08)	0.7
BMI (kg/m^2^)	25.8 (4.3)^a^	26.1 (3.8)	25.6 (4.67)	0.1
MMSE score	29.1 (1.3)	28.9 (1.43)	29.2 (1.12)	**0.01**
**Demographics:** *n* **(%)**				
*APOE ε4* carriership	225 (33.3%)	113 (34.6%)	112 (32.2%)	0.5
Current smokers	47 (7%)	22 (6.7%)	25 (7.2%)	0.8
Lifetime smokers	408 (60.4%)	201 (61.5%)	207 (59.5%)	0.6
Hypercholesterolaemia	401 (59.4%)	183 (56%)	218 (62.6%)	0.08
Hypertension	386 (57.2%)	190 (58.1%)	196 (56.3%)	0.6
Diabetes	59 (8.7%)	37 (11.3%)	22 (6.3%)	**0.03**
Atrial fibrillation	49 (7.3%)	31 (9.5%)	18 (5.2%)	**0.04**
**Brain MRI measures: mean (SD)**				
Total GM volume	581k (49k)	612k (42k)	552k (37k)	**2e-16**
Subcortical volume	53k (4k)	56k (4k)	51k (3k)	**2e-16**
Total WM volume	442k (52k)^a^	469k (47k)	417k (42k)	**2e-16**
Total WMHs volume	6.64 (8.58)^a^	7.98 (9.90)	5.37 (6.91)	**9e-05**
Log_10_ total WMHs volume	1.34 (1.08)	1.50 (1.14)	1.19 (1.00)	**1e-03**
Mean cortical thickness	2.35 (0.15)	2.34 (0.08)	2.36 (0.07)	**0.01**
Mean FA WM fibre tracts	0.45 (0.02)^b^	0.46 (0.02)	0.46 (0.02)	0.1
Total ICV	1507k (158k)	1611k (136k)	1410k (108k)	**2e-16**
**Vascular factors:** *n* **(%)**				
Binary SVD score	81 (12%)	50 (15.3%)	31 (8.9%)	**0.01**
Binary SVD score without WMHs	51 (7.6%)	30 (9.2%)	21 (6%)	0.1
Raw SVD score				
0	420 (62.2%)	201 (61.5%)	219 (62.9%)	0.06
1	174 (25.8%)	76 (23.2%)	98 (28.2%)	
2	56 (8.3%)	33 (10.1%)	23 (6.6%)	
3	23 (3.4%)	16 (4.9%)	7 (2%)	
4	2 (0.3%)	1 (0.3%)	1 (0.3%)	
WMHs on Fazekas scale	98 (14.6%)^c^	51 (15.6%)	47 (13.6%)	0.5
Cerebral microbleeds	71 (10.6%)^d^	31 (9.5%)	40 (11.6%)	0.5
EPVS in basal ganglia	147 (21.8%)^e^	83 (25.5%)	64 (18.4%)	**0.03**
Lacunes	47 (7%)	29 (8.9%)	18 (5.2%)	0.07
**Polygenic scores (PGSs): median (IQR)**				
*APOE* PGLS (Deelen et. al., 2019)	0.028 (1.36)	−0.007 (1.37)	0.051 (1.36)	0.4
Non-*APOE* PGLS (Deelen et. al., 2019)	0.042 (1.35)	−0.013 (1.37)	0.086 (1.35)	0.4

Total GM, WM and ICV volumes = mm^3^; Mean cortical thickness = mm, Total WMHs volume = millilitres (ml), Lacunes = 3–15 mm, Cerebral microbleeds = 2–5 mm, Participants in the Raw SVD score group 3 and 4, were classified into one group (23 + 2 = 25) to compute the *p*-value using Chi-squared test, WMHs on Fazekas scale were grouped as; none or single punctate = 0, multiple punctate = 1, early confluent = 2 and large confluent = 3 and the SVD score after excluding WMHs ranged from 0 to 3 (low burden = 0–1; *n *= 624, high burden = 2–3; *n* = 51). The brain MRI volumes are not adjusted by ICV in Table 1. Missing data: *n*^a^ = 1–4, *n*^b^ = 28, *n*^c^ = 3, *n*^d^ = 3 and *n*^e^ = 2.

*N* total sample; *n* (%) subsample (percentage), SD standard deviation, BMI body mass index, kg/m^2^ kilograms per metres squared, MMSE mini-mental state examination, IQR interquartile range, *APOE* PGLS PGLS with *APOE* locus inclusive and *non*-*APOE* *PGLS* PGLS without the *APOE* locus^2^, *GM* grey matter, *WM* white matter, *SVD* small vessel disease, *WMHs* white matter hyperintensities, *EPVS* enlarged perivascular spaces, *ICV* intracranial volume, *FA* fractional anisotropy.

### Primary analyses

In the total sample, we did not observe any associations between the *APOE* PGLS and the primary outcomes that included total GM, WM, and subcortical volumes, mean cortical thickness, mean FA values, total WMHs volume, and total SVD score (sTables 3 and 4).

**Fig. 1:**
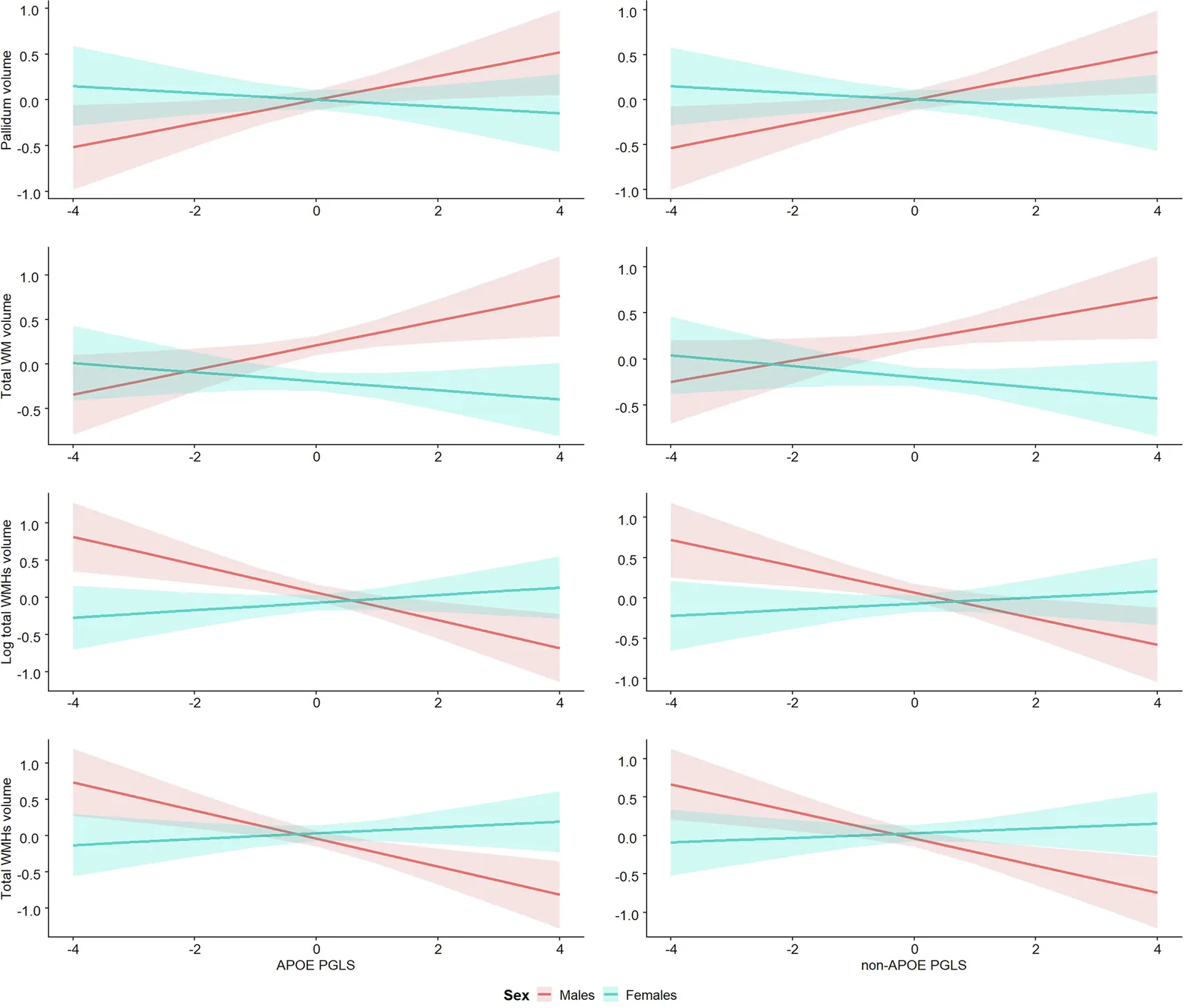
Interactions by sex in relation to the PGLSs and the brain MRI volumes in the total sample (*n* = 675).

**Fig. 2:**
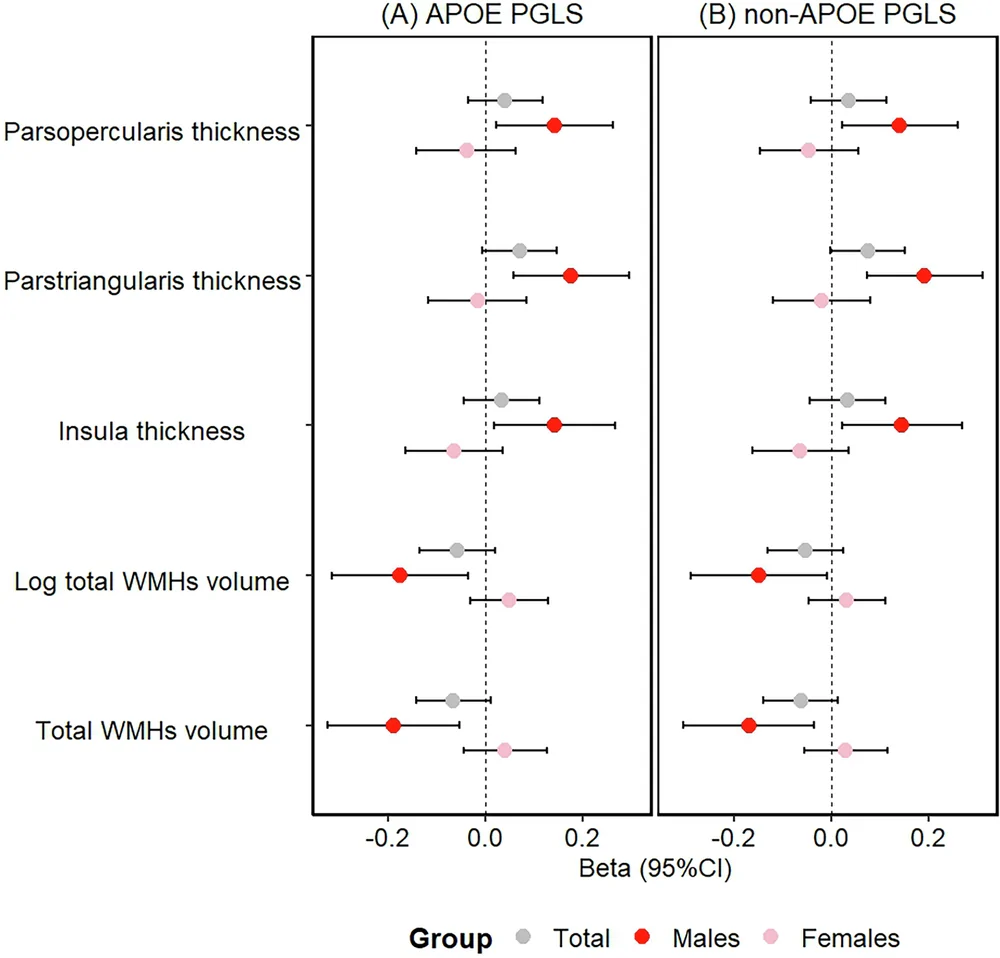
Associations between PGLSs and the brain MRI measures in the total sample (*n* = 675), males (*n* = 327), and females (*n* = 348). GM grey matter and WM white matter.

**Fig. 3:**
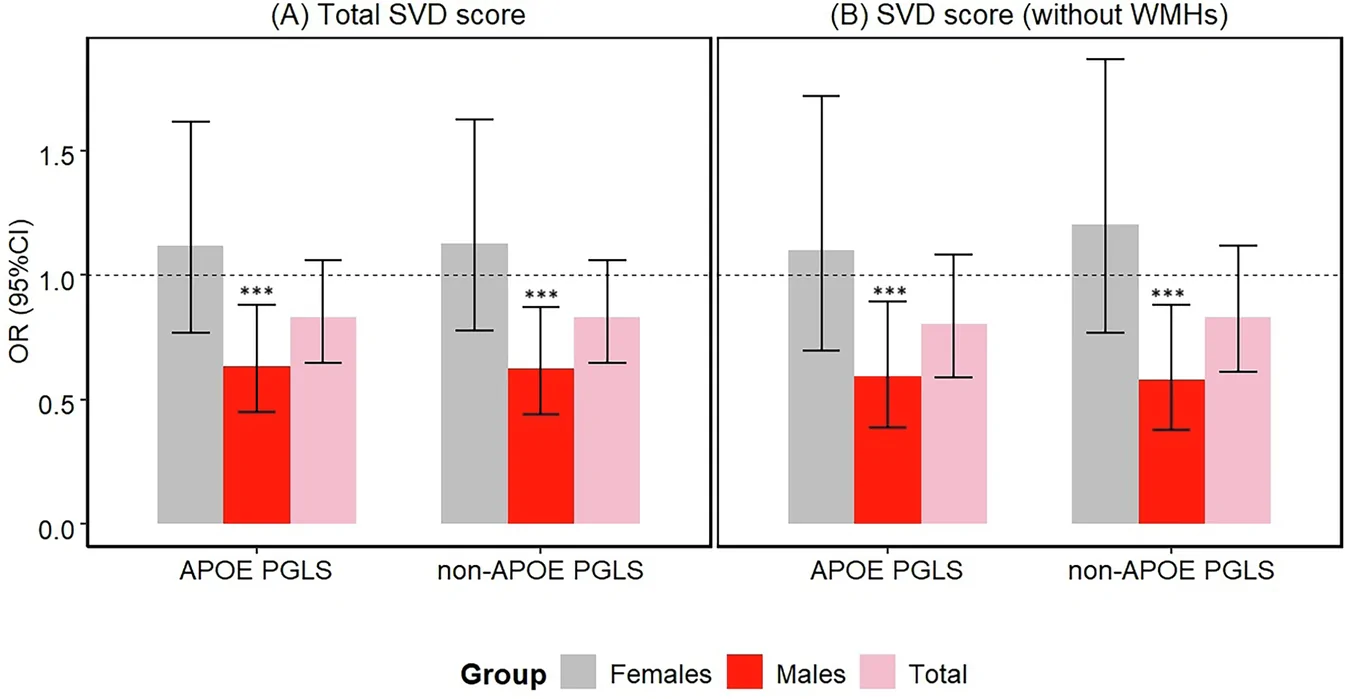
Associations between PGLSs and (**A**) total SVD score and (**B**) SVD score without WMHs in the total sample (*n* = 675), males (327), and females (*n* = 348). SVD small vessel disease, and WMHs white matter hyperintensities. The three black asterisks (***) represent significant results after correction for multiple testing.

We found significant interactions by sex in the relation between the *APOE* PGLS and total WM volume, total WMHs volume, and the total SVD score (sTable 5 and Fig. 1). Thus, among males, higher scores in the *APOE* PGLS were associated with smaller total WMHs volume (Beta = -0.18, *P*_corrected_ = 0.02), and lower total SVD burden (OR = 0.63, *P*_corrected_ = 0.01) (Table 2 and Figs. 2 and 3). No significant associations were observed among females, or between the *APOE* PGLS and total WM volume in sex-stratified analyses (sTable 6).

**Table 2:** Primary results: Sex-stratified associations between PGLSs and global MRI measures

Group	Brain measures	PGLSs	Beta/OR	LCI	UCI	*P*	*P*_corrected_
Males	Total WM volume	APOE PGLS	0.122	−0.005	0.250	0.0596	0.0596
Males	Total WMHs volume	APOE PGLS	−0.190	−0.326	−0.054	0.0062	**0.0185**
Males	Log Total WMHs volume	APOE PGLS	−0.176	−0.317	−0.035	0.0144	**0.0216**
Females	Total WM volume	APOE PGLS	−0.047	−0.135	0.041	0.2911	0.3605
Females	Total WMHs volume	APOE PGLS	0.040	−0.046	0.126	0.3605	0.3605
Females	Log Total WMHs volume	APOE PGLS	0.048	−0.032	0.128	0.2381	0.3605
Males	Total SVD score	APOE PGLS	0.632	0.449	0.880	0.0074	**0.0143**
Males	SVD score (without WMHs)	APOE PGLS	0.593	0.386	0.895	0.0143	**0.0143**
Females	Total SVD score	APOE PGLS	1.117	0.768	1.614	0.5564	0.6764
Females	SVD score (without WMHs)	APOE PGLS	1.100	0.697	1.718	0.6764	0.6764
Males	Total WM volume	non-APOE PGLS	0.098	−0.029	0.225	0.1292	0.1292
Males	Total WMHs volume	non-APOE PGLS	−0.170	−0.306	−0.035	0.0139	**0.0418**
Males	Log Total WMHs volume	non-APOE PGLS	−0.149	−0.290	−0.009	0.0376	0.0564
Females	Total WM volume	non-APOE PGLS	−0.054	−0.141	0.034	0.2274	0.4988
Females	Total WMHs volume	non-APOE PGLS	0.030	−0.056	0.115	0.4988	0.4988
Females	Log Total WMHs volume	non-APOE PGLS	0.032	−0.047	0.112	0.4230	0.4988
Males	Total SVD score	non-APOE PGLS	0.626	0.443	0.873	0.0065	**0.0120**
Males	SVD score (without WMHs)	non-APOE PGLS	0.582	0.377	0.881	0.0120	**0.0120**
Females	Total SVD score	non-APOE PGLS	1.129	0.779	1.626	0.5143	0.5143
Females	SVD score (without WMHs)	non-APOE PGLS	1.202	0.768	1.865	0.4140	0.5143

*PGLS* polygenic longevity score, *OR* odd ratio, *SVD* small vessel disease, *LCI* lower confidence limit, *UCI* upper confidence limit, and *P* *p* value.

In the total sample, we did not observe any associations between the non-*APOE* PGLS and the primary outcomes that included total GM, WM, and subcortical volumes, mean cortical thickness, mean FA values, total WMHs volume and total SVD score (sTables 3 and 4).

We found significant interactions by sex in the relation between the non-*APOE* PGLS and total WM volume, and the total SVD score (sTable 5 and Fig. 1). Thus, among males, higher scores in the non-*APOE* PGLS were associated with smaller total WMHs volume (Beta = -0.17, *P*_corrected_ = 0.04), and lower total SVD burden (OR = 0.63, *P*_corrected_ = 0.01) (Table 2 and Figs. 2 and 3). No significant associations were observed among females (sTable 6).

### Sensitivity analyses

In the total sample, we did not observe any associations between the *APOE* PGLS and the primary outcomes that included log total WMHs volume, SVD score without WMHs, and the raw SVD score(0-4) (sTables 3 and 4), and in the models adjusted for possible additional confounders (sTable 7).

We found significant interactions by sex in the relation between the *APOE* PGLS and log total WMHs volume (sTable 6 and Fig. 1). Thus, among males, higher scores in the *APOE* PGLS were associated with smaller log total WMHs volume (Beta = -0.19, *P*_corrected_ = 0.02) (Table 2 and Fig. 2). No significant associations were observed among females (sTable 7).

In the models adjusted for possible additional confounders, we found significant interactions by sex in the relation between the *APOE* PGLS and log total WMHs volume (sTable 8). No significant associations were, however, observed in sex-stratified analyses (sTable 9). In addition, we found no significant interactions by sex in the relation between the *APOE* PGLS and raw SVD score(0–4) (sTable 10).

In the total sample, we did not observe any associations between the non-*APOE* PGLS and the primary outcomes that included log total WMHs volume, SVD score without WMHs, and the raw SVD score(0-4) (sTables 3 and 4), and in the models adjusted for possible additional confounders (sTable 7).

We found significant interactions by sex in the relation between the non-*APOE* PGLS and log total WMHs volume, and the SVD without WMHs (sTable 6 and Fig. 1). Thus, among males, higher scores in the non-*APOE* PGLS were associated with lower SVD without WMHs burden (OR = 0.58, *P*_corrected_ = 0.01) (Table 2 and Figs. 2 and 3). No significant associations were observed among females, or between the non-*APOE* PGLS and total WMHs volume in sex-stratified analyses (sTable 7).

In the models adjusted for possible additional confounders, we found significant interactions by sex in the relation between the non-*APOE* PGLS and log total WMHs volume, and SVD score without WMHs (sTable 8). Thus, in males, higher non-*APOE* PGLS was associated with lower SVD score without WMHs (OR = 0.59, *P*_corrected_ = 0.03) (sTable 9). We did not observe any significant associations between the non-*APOE* PGLS and total WMHs volume in relation to the sex groups (sTable 9). No significant interactions by sex were observed in the relation between the non-*APOE* PGLS and the raw SVD score(0–4) (sTable 10).

### Secondary analyses

We did not observe any associations between the *APOE* PGLS and the secondary MRI outcomes that included the regional subcortical volumes, regional cortical thicknesses, and FA WM fibre tracts in the total sample (sTables 11, 12, 13).

**Fig. 4:**
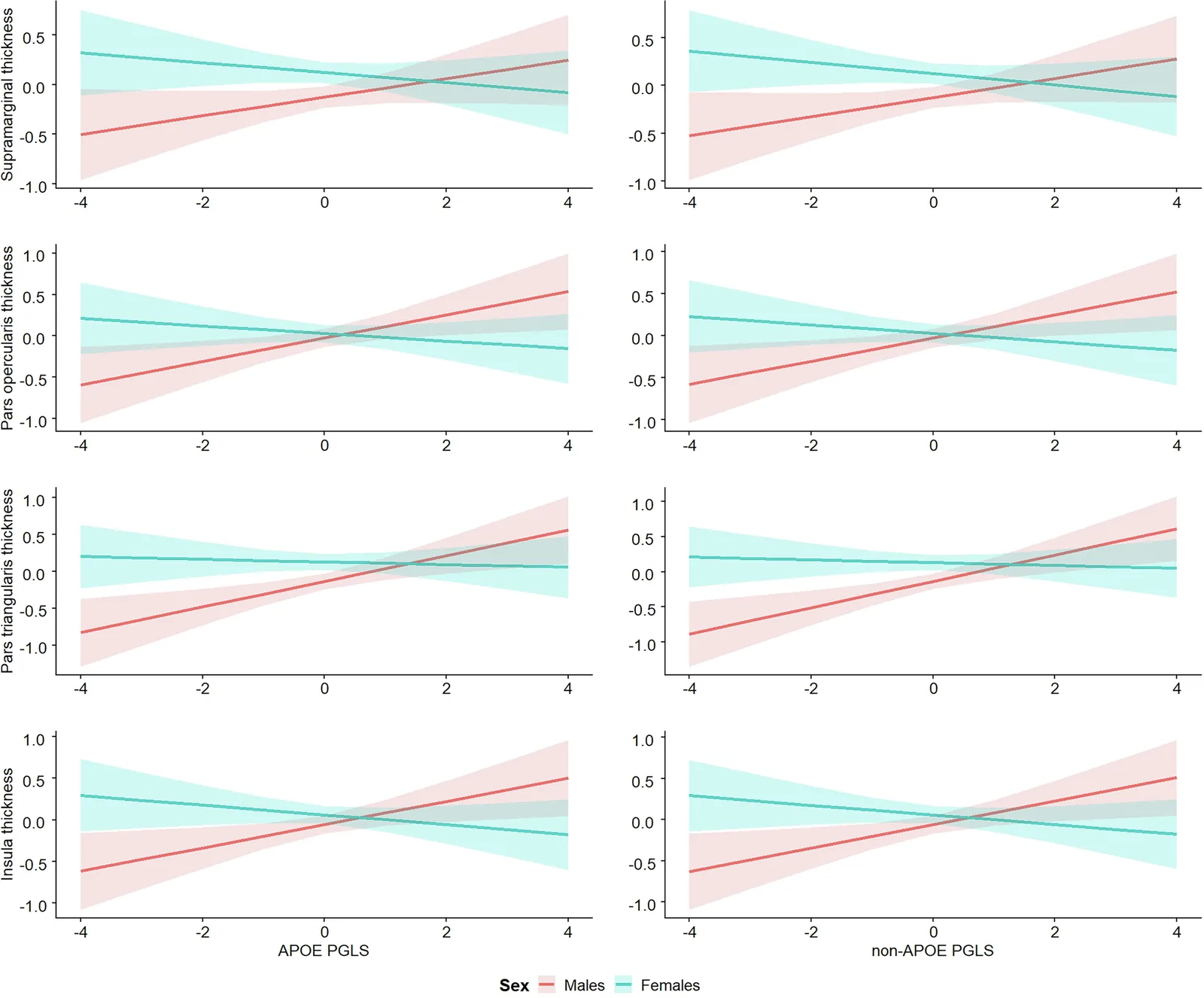
Interactions by sex in relation to the PGLSs and regional cortical thickness values in the total sample (*n* = 675).

We found significant interactions by sex in the relation between the *APOE* PGLS and volume of pallidum, and thicknesses of pars triangularis, pars opercularis, and insula (sTables 14 and 15 and Figs. 1 and 4). There were no interactions by sex in the relation with FA WM fibre tracts (sTable 16). Thus, among males, higher scores in the *APOE* PGLS were associated with thicker cortex of the pars triangularis (Beta = 0.18, *P*_corrected_ = 0.02), pars opercularis (Beta =  0.14, *P*_corrected_ = 0.03), and insula (Beta = 0.14, *P*_corrected_ = 0.03,) (sTable 17 and Fig. 2). We did not observe significant associations between the *APOE* PGLS and pallidum volume in the sex-stratified analyses (sTable 18).

We did not observe any associations between the non-*APOE* PGLS and the secondary MRI outcomes that included regional subcortical volumes, regional cortical thicknesses, and FA WM fibre tracts in the total sample (sTables 11, 12, 13).

We found significant interactions by sex in the relation between the non-*APOE* PGLS and pallidum volume, and pars triangularis, pars opercularis, insula and supramarginal thicknesses insula (sTables 14 and 15 and Figs. 1 and 4). There were no interactions by sex in relation to the FA WM fibre tracts (sTable 16). In the sex-stratified analyses, among males, higher scores in the non-*APOE* PGLS were associated with a thicker cortex of the pars triangularis (Beta = 0.19, *P*_corrected_ = 0.007), pars opercularis (Beta = 0.14, *P*_corrected_ = 0.03), and insula (Beta = 0.14, *P*_corrected_ = 0.03) (sTable 17 and Fig. 2). However, we did not observe significant associations between the non-*APOE* PGLS and the supramarginal thickness (sTable 17) and pallidum volume (sTable 18) in the sex-stratified analyses.

## Discussion

In this population-based study, we found that 70-year-old males with higher scores in our primary exposure (*i.e.*, *APOE* PGLS) had fewer total WMHs, lower total SVD burden (primary outcomes), and thicker cortex of the pars triangularis, pars opercularis, and insula (secondary outcomes), after correction for multiple comparison tests. This was also the case for the non-*APOE* PGLS (secondary exposure). These sex-specific findings were supported by the interaction analyses. There were no associations in the total sample, and the findings must therefore be interpreted carefully until confirmed in other studies (Tables 3–5).

**Table 3:** Primary results: Sex-stratified associations between PGLSs and vascular factors adjusted for possible confounders

Group	Brain measures	PGLSs	Beta/OR	LCI	UCI	*P*	*P*_corrected_
Males	Total WMHs volume	APOE PGLS	−0.061	−0.139	0.015	0.1193	0.1193
Females	Total WMHs volume	APOE PGLS	−0.061	−0.139	0.015	0.1193	0.1193
Males	Log Total WMHs volume	APOE PGLS	−0.057	−0.134	0.021	0.1465	0.1465
Females	Log Total WMHs volume	APOE PGLS	−0.057	−0.134	0.021	0.1465	0.1465
Males	Total SVD score	APOE PGLS	0.698	0.482	0.999	0.0523	0.0523
Females	Total SVD score	APOE PGLS	1.075	0.733	1.571	0.7068	0.7068
Males	SVD score (without WMHs)	APOE PGLS	0.624	0.388	0.982	0.0454	0.0454
Females	SVD score (without WMHs)	APOE PGLS	1.037	0.642	1.662	0.8792	0.8792
Males	Total WMHs volume	non-APOE PGLS	−0.061	−0.138	0.015	0.1188	0.1188
Females	Total WMHs volume	non-APOE PGLS	−0.061	−0.138	0.015	0.1188	0.1188
Males	Log Total WMHs volume	non-APOE PGLS	−0.055	−0.131	0.023	0.1641	0.1641
Females	Log Total WMHs volume	non-APOE PGLS	−0.055	−0.131	0.023	0.1641	0.1641
Males	Total SVD score	non-APOE PGLS	0.674	0.464	0.968	0.0352	**0.0352**
Females	Total SVD score	non-APOE PGLS	1.079	0.736	1.575	0.6923	0.6923
Males	SVD score (without WMHs)	non-APOE PGLS	0.591	0.363	0.936	0.0284	**0.0284**
Females	SVD score (without WMHs)	non-APOE PGLS	1.134	0.708	1.808	0.5962	0.5962

*PGLS* polygenic longevity score, *OR* odd ratio, *SVD* small vessel disease, *LCI* lower confidence limit, *UCI* upper confidence limit, and *P* *p* value.

**Table 4:** Secondary results: Sex-stratified associations between PGLSs and subcortical volumes

Group	Brain measures	PGLSs	Beta	LCI	UCI	*P*	*P*_corrected_
Males	Pallidum volume	APOE PGLS	0.108	−0.017	0.233	0.0896	0.0896
Females	Pallidum volume	APOE PGLS	−0.039	−0.136	0.058	0.4258	0.4258
Males	Pallidum volume	non-APOE PGLS	0.111	−0.014	0.235	0.0808	0.0808
Females	Pallidum volume	non-APOE PGLS	−0.038	−0.135	0.058	0.4329	0.4329

*PGLS* polygenic longevity score, *LCI* lower confidence limit, *UCI* upper confidence limit, and *P* *p* value.

**Table 5:** Secondary results: Sex-stratified associations between PGLSs and regional cortical thickness

Group	Brain measures	PGLSs	Beta	LCI	UCI	*P*	*P*_corrected_
Males	Pars opercularis thickness	APOE PGLS	0.143	0.023	0.263	0.0197	**0.0328**
Males	Pars triangularis thickness	APOE PGLS	0.176	0.057	0.296	0.0040	**0.0161**
Males	Insula thickness	APOE PGLS	0.142	0.018	0.266	0.0246	**0.0328**
Males	Supramarginal thickness	APOE PGLS	0.084	−0.036	0.204	0.1706	0.1706
Females	Pars opercularis thickness	APOE PGLS	−0.039	−0.142	0.063	0.4501	0.6001
Females	Pars triangularis thickness	APOE PGLS	−0.017	−0.119	0.084	0.7411	0.7411
Females	Insula thickness	APOE PGLS	−0.065	−0.165	0.035	0.2039	0.6001
Females	Supramarginal thickness	APOE PGLS	−0.045	−0.146	0.056	0.3766	0.6001
Males	Pars opercularis thickness	non-APOE PGLS	0.141	0.022	0.261	0.0208	**0.0278**
Males	Pars triangularis thickness	non-APOE PGLS	0.192	0.073	0.311	0.0017	**0.0067**
Males	Insula thickness	non-APOE PGLS	0.146	0.022	0.269	0.0206	**0.0278**
Males	Supramarginal thickness	non-APOE PGLS	0.093	−0.027	0.212	0.1292	0.1292
Females	Pars opercularis thickness	non-APOE PGLS	−0.046	−0.147	0.056	0.3796	0.5061
Females	Pars triangularis thickness	non-APOE PGLS	−0.020	−0.121	0.081	0.6996	0.6996
Females	Insula thickness	non-APOE PGLS	−0.064	−0.163	0.035	0.2062	0.5061
Females	Supramarginal thickness	non-APOE PGLS	−0.056	−0.156	0.044	0.2735	0.5061

*PGLS* polygenic longevity score, *LCI* lower confidence limit, *UCI* upper confidence limit, and *P* *p* value.

Our finding of an association between higher PGLSs and lower total WMHs volume in males is in line with a previous study, reporting that higher PGLSs (both the primary and secondary exposures) were associated with lower WMHs volume in males but not in females^18^. Our study is the first to examine associations between PGLSs and total SVD score, which is biologically plausible as vascular brain diseases are established risk factors for mortality^11,12^.

The finding that all observed associations were in males only is in line with the other study, which examined PGLSs in relation to brain MRI findings^18^. The reason for our findings of different results for males and females might be multifold. First, males had higher vascular burden and more WMHs compared to females (Table 1), and the variation in WMHs was higher in males than in females. This might be one reason why we only found associations between higher PGLS and lower vascular burden in males. Second, as we only examined 70-year-olds, we do not know if the association in females will appear later in life. Females live longer than males^25^, and might have a lower biological age than males at this point in life^26^. Third, factors related to survival may differ by sex. Our PGLSs may be less sensitive in females or more sensitive in males. For example, deficiency or loss of the male-specific Y chromosome, associated with age-related diseases and shortened lifespan^27,28^, is common in older males (8.6–57%). The lower survival rate in males is also suggested to be related to a higher rate of vascular risk factors, leading to an earlier onset of ischaemic heart disease^25^. Thus, males with these factors may be more vulnerable to brain changes if they have a lower PGLSs. Fourth, peripheral oestrogen and loss of it during menopause might play a role, e.g. studies report that a longer reproductive period is related to decreased total brain volume^29^, and increased risk for dementia and biomarkers of AD^30,31^, and higher estradiol levels to smaller hippocampal volume^32^. Fifth, males had larger brains and more variations in brain size (*i.e.*, larger intracranial volume reported in Table 1), which might make it easier to find associations among males compared with females.

Interestingly, our findings were not exclusively driven by contributions from the *APOE* locus, which is in line with the only study on this matter^18^. This also highlights the importance of other longevity-related genes than the *APOE* locus^2^ for brain structures. In our sensitivity analyses, after correction for vascular and lifestyle-related factors (years of education, smoking, hypertension, BMI, diabetes and hypercholesterolaemia), the results were attenuated for some of the primary vascular outcomes (the SVD score, total WMHs volume and log total WMHs volume). However, the association between the non-*APOE* PGLS and the total SVD score and SVD without WMH remained significant in males and may suggest that the associations with the *APOE* PGLS is more related to vascular risk factors, while the non-*APOE* PGLS may partly have other pathways to these brain changes.

Regarding the SVD score, we excluded WMHs from the score in the sensitivity analyses because WMHs is the most prevalent component of the score and might have been the driver of the associations. The associations remained, however, largely unchanged after excluding WMHs from the total SVD score (*i.e.*, both the interactions and male sex-specific associations). This suggests that the findings were not solely driven by WMHs but may reflect a broader association with SVD burden. However, the total WMHs volume showed a right-skewed distribution (Table 1). We therefore evaluated its robustness by computing the log-transformed total WMHs volume. The direction and magnitude of the association for the *APOE* PGLS were broadly consistent in both the total WMHs volume and log total WMHs volume, supporting the robustness of the finding. However, the association for the non-*APOE* PGLS was no longer statistically significant after the log-transformation of the total WMHs volume, which may indicate an influence by participants with high lesion burden. i.e. the associations are not continuous but rather on a threshold level.

The lack of associations between PGLSs and total GM volumes was unexpected, as several studies report that smaller cortical volumes and cortical atrophy on MRI are related to mortality^11,12^. Our PGLSs may act on longevity via pathways not affecting brain volume. Other explanations for the lack of associations include the sample size, the relatively young and homogeneous age of our sample and the exclusion of stroke, dementia and some neurological disorders.

In the secondary MRI outcome analyses, the only associations with GM area in our study after correction for multiple comparisons were associations between both PGLSs, and pars triangularis, pars opercularis, and insula thicknesses in males, findings which have not been reported previously. The findings on the pars triangularis and pars opercularis are interesting as these regions are both located in the inferior frontal gyrus of Broca’s region, and this region plays a critical role in mediating language production. The pars triangularis is critical for the semantic processing of both spoken and written language^33^. The pars opercularis is involved in processing speech sounds, building grammatically correct sentences, and planning the movements needed for speech^34^. The insula is located deep within the lateral sulcus, and is involved in a number of functions important for survival^35,36^, *e.g*., regulation of body homoeostasis, processing of sensory information from the body, respiratory regulation, feelings of hunger and pain^37–39^, and also vulnerable to neurodegenerative diseases^40^. It is important to mention that the only other study on this matter did not find associations between PGLSs and any GM volume, including the hippocampus, but they did not examine pars triangularis, pars opercularis, and insula^18^. Thus, this finding has to be taken cautiously until confirmed in other studies.

Among the strengths of this study are the comprehensive examinations of a relatively large sample of age-homogeneous septuagenarians from the general population with both genetic and MRI data. There are also some limitations. First, we adjusted for multiple testing to reduce the chance of type I errors. As discussed above, we may therefore have missed some true associations, which potentially might cause type II errors. We therefore decided to also include results that disappeared after correction for multiple comparisons, as suggested by others^41^. We take note of the fact that all the findings that did not survive correction for multiple comparisons in our study are biologically plausible and all are in the expected direction, but further confirmations are needed from future studies. Although we did correction for multiple comparisons, it is important to note that each MRI outcome represents a separate biological question and should be interpreted individually rather than being penalised because several outcomes were analysed. We therefore emphasise that the results should be interpreted in the context of the hypotheses, biological plausibility, consistency with previous evidence, effect sizes, and confidence intervals rather than only relying on statistical significance metrics after multiple comparison tests. Second, although the response rate was fairly high, we cannot exclude the possibility that there were important differences between participants and non-responders that might have affected our results. In addition, we excluded individuals with dementia, stroke, and several neurological disorders, and the MRI sample was slightly healthier than those who did not undergo an MRI. All these factors probably made it more difficult to find associations with the PGLSs. Third, participants are age-homogeneous and of European ancestry, limiting the possibility to generalise to other ages and ethnic groups. Fourth, it needs to be emphasised that the PGLSs capture only a fraction of the variance in human longevity. In addition, the PGLSs include genes involved in pathways that modulate age-related diseases/or traits^1,2^, and these are also related to changes in the brain and mortality. This might explain the strong associations with vascular burden, but it is surprising that we did not find more associations with structural brain changes in our study. Fifth, interpretations from cross-sectional studies should be taken cautiously, but genetic risk factors are supposed to be present since birth, and reversed causality should not be present. Sixth, a selective survival bias might have influenced the results. One could expect that in a study of 70-year-olds, those who survived to age 70 might be healthier than those who died, which makes it less possible to find differences between the groups. However, in this Swedish birth cohort born in 1944, 76% of males and 83% of females survived to age 70. Seventh, in support of our findings, there are also other basic performance/context for PGLSs in this cohort. A recent study in this cohort reported that higher PGLS was associated with lower biological brain age in 70-year-old males^42^. In another study using participants in the present study (manuscript resubmitted), higher PGLSs were associated with less hypertension, fewer myocardial infarctions, higher educational level, and better childhood and current socioeconomic status. However, the PGLSs were not associated with parental longevity. Eighth, the SVD score was dichotomised into a binary variable due to a few observations in the higher SVD burden group. This approach may lead to loss of information as participants with different levels of SVD burden are treated as equivalent, ignoring the ordered nature of the SVD severity. To evaluate the robustness of our findings on the SVD score, we therefore performed a sensitivity analysis treating the SVD score as an ordinal outcome(0-4). The association was no longer statistically significant. One possible explanation could be that our observed findings may depend on the chosen modelling strategy. Alternatively, this may reflect reduced statistical power due to sparse observations in the higher SVD burden group, or that the associations with the SVD scores are not on a continuous but rather on a threshold level.

In conclusion, in a population sample of 70-year-olds, our primary findings are that higher scores in the PGLSs were associated with smaller total WMHs volume, and lower total SVD burden in males, but not in females. In secondary analyses, we found that in males, higher PGLSs were associated with a thicker cortex of the pars triangularis and pars opercularis (brain regions involved in language production) and insula (brain region involved in the regulation of body homoeostasis). This was the case for PGLSs both with and without the inclusion of the *APOE* locus, highlighting the importance of genes other than the *APOE* locus for brain structures. The findings suggest a possible link between genetic propensity for longevity and lower cerebrovascular brain injury burden in males in this cohort. From a broader public health context, it further supports the importance of detection and treatment of vascular disorders and risk factors to promote healthy brain ageing and longevity in human populations.

## Methods

### Study population

**Fig. 5:**
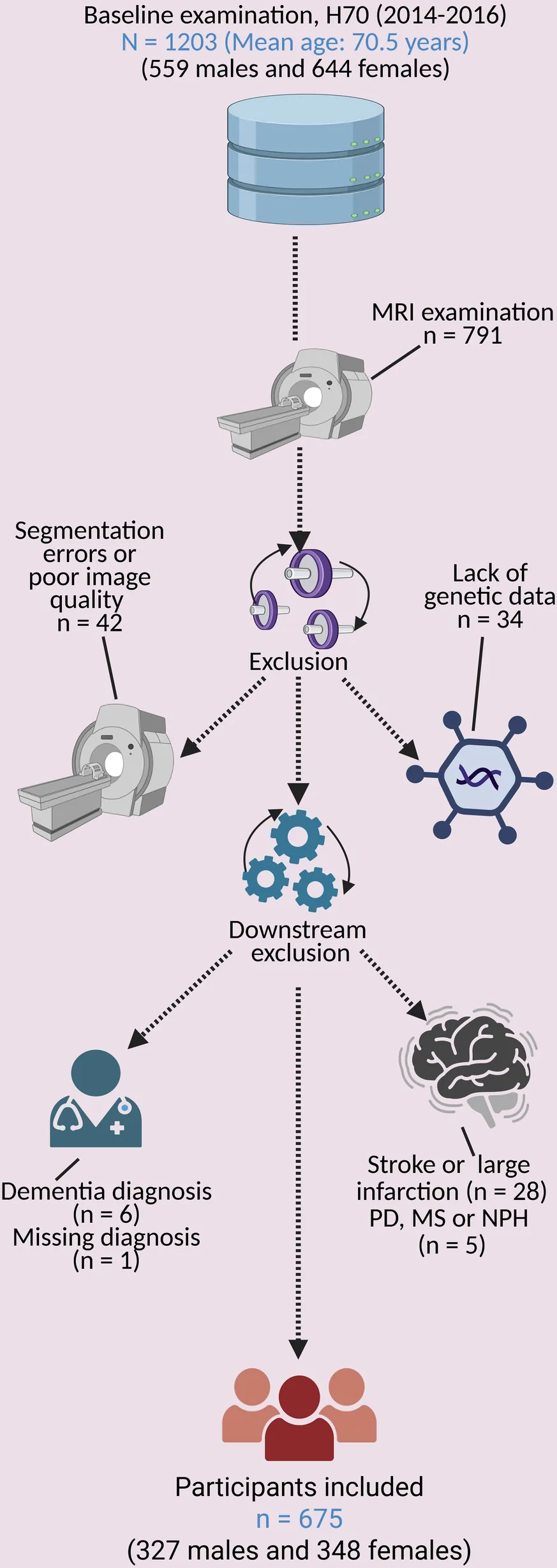
Flowchart of the inclusion of participants for the study. MRI magnetic resonance imaging, PD Parkinson’s disease, MS multiple sclerosis and NPH normal pressure hydrocephalus. Created in BioRender. Seidu, N. (2026) https://BioRender.com/a88m391.

In 2014, using the Swedish Tax Agency’s register, all 70-year-olds born in 1944 on birthdates ending with 0, 2, 5 and 8, and living in Gothenburg were invited to the H70 Birth Cohort 1944 Study. Full details of the study are described elsewhere^43^. Of the 1203 70-year-olds (response rate 72%) that took part in the study, 791 individuals (377 males and 414 females) underwent MRI (Fig. 5). Those with an MRI had higher education, less often dementia, and were less often smokers compared to those without an MRI, as described previously^44^. In addition, there were no differences between participants with or without MRI regarding the PGLSs. We excluded 116 individuals: 42 failed MRI quality control (QC), 34 lacked genetic data, 28 due to stroke or large infarction, five due to neurological disorders, six due to dementia and one due to missing data on dementia, leaving 675 participants. Among these, 28 had missing data on diffusion MRI (dMRI), but were included in all other MRI analyses.

### Ethical approvals and informed consents

The Regional Ethical Review Board at Västra Götaland Region, Gothenburg, Sweden, approved the study (Dnr 869-13, Dnr 06-14, and T703/14). Importantly, written informed consent, in accordance with the Declaration of Helsinki, was obtained from all participants or from their relatives if the participant was not able to provide consent. Information regarding voluntary participation and the freedom to interrupt the examination at any time without giving reasons for it, or only take part in some of the examinations, is given in the invitation letter, and before the actual examinations. Clinical trial number: not applicable.

### Data collection

Research nurses or medical doctors conducted the general examination and interviewed participants for information regarding social factors (*e.g.*, education), lifestyle factors (*e.g.*, smoking), medical conditions and medications^43^. Physical examinations (*e.g.*, height, weight, systolic and diastolic blood pressure), and blood tests were conducted^43,44^. Neuropsychiatric examinations included semi-structured interviews and cognitive tests conducted by experienced psychiatric nurses and medical doctors^43^. Register data were collected from the national patient register (NPR) classified according to the International Classification of Diseases, tenth edition-Sweden (ICD-10-SE). Close informant interviews were conducted by telephone by research nurses or a psychologist^43^.

Dementia diagnoses were used for exclusion and based on the Diagnostic and Statistical Manual of Mental Disorders Third Edition-Revised (DSM-III-R) using information from neuropsychiatric examinations and close informant interviews, as described elsewhere^45^. Stroke diagnoses were based on self-reports, close informant interviews, and register data from the NPR^44,46^. Diagnoses of Parkinson’s disease, multiple sclerosis, and normal pressure hydrocephalus were based on self-reports and the NPR, and atrial fibrillation (AF) was based on self-report, ECG and the NPR^44^. Hypercholesterolaemia was defined as present treatment with lipid-lowering drugs or total cholesterol >6.2, LDL ≥4.1, triglyceride >5.6, or HDL <1.0 (in males) or <1.3 (in females). Hypertension was defined as systolic blood pressure ≥150 or diastolic blood pressure ≥90, or the use of antihypertensive medication, and diabetes mellitus as ongoing treatment with insulin or antidiabetic drug, fasting glucose ≥7.0 mmol/L, or nonfasting glucose ≥11.1 mmol/L, as described previously^44^.

### Genotyping and imputation

DNA from whole blood was extracted using standard procedures, and genotyping was performed using the NeuroChip from Illumina^43^. Prior to imputation, QC was performed on single-nucleotide polymorphisms (SNPs)^46^. Variants were excluded if they had a call rate <0.98, a missingness difference between groups >0.02, a minor allele frequency (MAF) <0.01, or deviated from Hardy–Weinberg equilibrium (HWE), *P* < 1e-6^46^. The Haplotype Reference Consortium (GRCh37) panel was used on the genotype data to compare SNP IDs, strand, and allele frequencies^46^. We used the Sanger imputation service to impute the high-quality controlled autosomal variants^46^. We used PLINK software for post-imputation QC, excluding variants with MAF <0.01, HWE (*P* < 1e-6), and excluded SNPs with imputation INFO scores ≤0.8 provided by the Sanger Imputation Service, resulting in ~7 million high-quality autosomal SNPs.

### Construction of the PGLSs

The PGLSs were generated using the Deelen et al. (2019) GWAS summary statistics on human longevity (Accession number: GCST008598)^2^, which did not include participants in this present study. The PRS-CS method, which is a Bayesian regression framework^3^, was used to infer posterior SNP effect sizes by using default parameter settings. This method applies a continuous shrinkage prior that adaptively shrinks SNP effect sizes according to their GWAS association strength and local LD structure, determined using an external LD reference panel (1000 Genomes European, GRCh37)^3^. Briefly, the GWAS longevity phenotype cases were individuals who lived to age above the 90th percentile for survival (*n* = 11,262), and controls were individuals who died at or before the corresponding 60th percentile (*n* = 25,483)^2^. To account for the strong influence of the *APOE* locus on survival^2^. We constructed two polygenic scores^1^; an *APOE*-inclusive PGLS and^2^ a non-*APOE* PGLS (excluding variants in the *APOE* locus; chromosome 19, coordinates GRCh37: 44412079–46412079). This was done as the *APOE* locus is strongly related to shorter survival^2^. For clarity, a higher PGLS value reflects greater genetic propensity for longevity.

### MRI acquisition

The participants were scanned on a 3T Philips Achieva system at one site. Detailed imaging acquisition protocols for the T1-weighted, T2-weighted, FLAIR, dMRI, and susceptibility weighted imaging (SWI) are shown in sTable1. The MRI images are managed and stored in a web-based brain-imaging database, and detailed imaging and post-reconstruction QC were adapted according to previously published criteria^47^.

### MRI analyses

The FreeSurfer version 7.2.0 was used to generate the volumes and perform cortical reconstructions. Total GM and WM volumes, as well as subcortical volumes (hippocampus, amygdala, caudate, thalamus, putamen, pallidum and accumbens) were adjusted for intracranial volume (ICV)^48^. The cerebral cortex was automatically delineated into 34 standard gyral-based ROIs (Desikan atlas), and thickness measures were extracted for each ROI in each hemisphere.

The total WMHs volume was automatically segmented using the LST v2.0.15 for SPM^49^, and adjusted for ICV. For the dMRI processing, we used the ICBM-DTI-81 atlas to generate and label 48 FA fibre tracts using the FDT software^50^. The individual FA maps were registered to the MCALT standard atlas using the ANTs toolbox. Because of the greater sensitivity of FA to subtle WM microstructural alterations relative to other dMRI-derived metrics in older adults, we focus on the FA fibre tracts^10^. To simplify interpretation, we averaged the right and left hemispheres of the MRI measures (regional subcortical volumes, regional cortical thickness, and FA WM fibre tracts values)^51^.

The total SVD score was rated by a radiologist according to STRIVE criteria and included lacunes, cerebral microbleeds (CMBs), enlarged perivascular spaces (EPVS) in the basal ganglia, and WMHs on the Fazekas scale, described elsewhere^52^. We created the total SVD score by first assigning one point to the presence of each of these four MRI features. The total SVD score was grouped into a binary variable (low burden = 0–1; high burden = 2–4) due to very few individuals with high burden. An additional SVD score was calculated after excluding WMHs (low burden = 0–1; high burden = 2–3).

### Statistical analyses

Sex differences in demographics were tested with Fisher’s exact test for categorical variables and an independent sample *t*-test for means. All continuous variables were z-transformed. All longevity models were adjusted for age (age range at baseline MRI was 70–73 years) and the first ten principal components to account for population stratification due to the genetic data distribution. Models not stratified by sex were adjusted for sex.

In the primary analyses, the associations between our primary exposure (*APOE* PGLS) and our primary MRI outcomes were tested using linear regression models (for total GM, WM, WMHs and subcortical volumes, mean cortical thickness, and mean FA values) or logistic (total SVD score) in the total sample and stratified by sex if sex interactions were significant. We tested sex interactions because sex differences in human brain anatomy and human longevity are well-documented^53,54^. Analyses for the primary MRI outcomes were repeated using the secondary exposure (non-*APOE* PGLS). In sensitivity analyses, we tested the relation between the *APOE* PGLS and log total WMHs volume (to assess model diagnostics that may result from a highly skewed distribution of WMHs) using linear regression, SVD score after excluding WMHs on the Fazekas scale (SVD score without WMHs) using logistic regression, and the raw SVD score (range 0 = low burden to 4 = high burden) using an ordinal regression model. In addition, the analyses for the total WMHs and the total SVD score were repeated by adjusting for possible vascular and lifestyle confounders (years of education, smoking, hypertension, BMI, diabetes, and hypercholesterolaemia). We performed these analyses to assess whether these factors influence the direct effect size of the exposure on the outcome. It is important to highlight that these vascular/lifestyle factors should be treated as mediators of the associations, i.e. the PGLSs might cause these factors or mediators, but the lifestyle factors are not likely to affect the PGLSs.

In the secondary analyses, we examined the primary exposure (*APOE* PGLS) in relation to the regional subcortical volumes, regional cortical thickness, and the FA fibre tracts values. These associations were tested using linear regression models in the total sample and stratified by sex if sex interactions were significant. Analyses for the secondary MRI outcomes were repeated using the secondary exposure (non-*APOE* PGLS).

For the SVD analyses, we report odds ratios (ORs) and confidence intervals (CIs). For the total GM, WM, WMHs and subcortical and regional subcortical volumes, regional cortical thickness, and FA in WM fibre tracts values, we report standardised Beta (β) coefficients and CIs. All *P* values (*P*) for the association analyses were corrected (*P*_corrected_) for multiple comparisons using the Benjamini–Hochberg false discovery rate^55^, and this was applied separately within each MRI outcome. Specifically, corrections were applied within each predefined MRI outcome for each exposure (*APOE* PGLS and non-*APOE* PGLS) and sex-specific analysis separately. A similar approach was applied in the secondary MRI outcome analyses.

## Supplementary information

Supplementary Information

## Data Availability

The data that support the findings of this study are not publicly available because the study protocol did not include a provision for data to be shared publicly. However, data request approval is required to have access and will be made available in accordance with the General Data Protection Regulation (GDPR) upon reasonable request. Please contact the nazib.m.seidu@gu.se, ingmar.skoog@neuro.gu.se or eric.westman@ki.se. We used readily available open software for all analyses in this project.

## Code availability

Data processing and statistical analyses were performed in Jupyter Notebook 6.5.4 and RStudio 4.4.0 and are available from the corresponding author upon reasonable request.
