# Supplementary material for: Polygenic scores for longevity in relation to brain MRI measures in a cohort of 70-year-olds
Source: NPJ Aging. 2026 Oct 15;12(1):144. doi: 10.1038/s41514-026-00546-8 (PMC13672337; doi:10.1038/s41514-026-00546-8)
Supplement: Supplementary file 1 [file 41514_2026_546_MOESM1_ESM.pdf]

SUPPLEMENTARY MATERIALS

sTable 1: Details of the MRI image acquisition protocol at age 70 examination.

Sequence	T1-weighted	T2-weighted	FLAIR	dMRI	SWI
Repetition time (ms)	7.2	3000	4800	7340	14-18
Echo time (ms)	3.2	80	280	83	20-26
Field of view (mm)	256 x 256	230 x 230	250 x 250	224 x 224	220 x 220
Matrix (mm)	250 x 250	288 x 223	250 x 237	112 x 112	220 x 222
Flip angle (°)	9	90	90	90	10
Slice thickness (mm)	1	4	2	3	1

FLAIR = Fluid-Attenuated Inversion Recovery, dMRI = diffusion magnetic resonance imaging and SWI = susceptibility weighted imaging.

sTable 2: The number of SVD events by sex and PGLSs

Variable	Total	No SVD	SVD Events	P-value
**Sex: n (%)**				
Male	327 (48.4%)	277 (46.6%)	50 (61.7%)	**0.012**
Female	348 (51.6%)	317 (53.4%)	31 (38.3%)	
**Polygenic scores (PGSs): median (IQR)**				
APOE PGLS (Deelen *et. al.*, 2019)	0.028 (1.36)	0.051 (1.36)	-0.174 (1.12)	0.2
Non-*APOE* PGLS (Deelen *et. al.*, 2019	0.042 (1.35)	0.084 (1.38)	-0.221 (1.32)	0.2

SVD = small vessel disease, *APOE* PGLS = PGLS with the *APOE* locus inclusive, and non-*APOE* PGLS = PGLS without the *APOE* locus.

sTable 3: Primary results - Association between PGLSs and global MRI measures in the total sample

Brain MRI measures	PGLSs	Beta/OR	LCI	UCI	P	Adjusted P
Total GM volume	APOE PGLS	0.011	-0.066	0.088	0.7851	0.7851
Subcortical volume	APOE PGLS	0.049	-0.017	0.116	0.1463	0.2128
Total WM volume	APOE PGLS	0.036	-0.039	0.111	0.3429	0.5844
Total WMHs volume	APOE PGLS	-0.067	-0.144	0.011	0.0908	0.1069
Log Total WMHs volume	APOE PGLS	-0.058	-0.136	0.019	0.1416	0.1753
Mean cortical thickness	APOE PGLS	0.009	-0.068	0.086	0.8143	0.8895
Mean FA values	APOE PGLS	0.002	-0.077	0.081	0.9610	0.9610
Total SVD score	APOE PGLS	0.830	0.648	1.059	0.1367	0.1373
SVD score (without WMHs)	APOE PGLS	0.802	0.591	1.083	0.1520	0.2230
Total GM volume	non-APOE PGLS	0.017	-0.060	0.093	0.6721	0.7851
Subcortical volume	non-APOE PGLS	0.042	-0.024	0.109	0.2128	0.2128
Total WM volume	non-APOE PGLS	0.021	-0.054	0.096	0.5844	0.5844
Total WMHs volume	non-APOE PGLS	-0.063	-0.141	0.014	0.1069	0.1069
Log Total WMHs volume	non-APOE PGLS	-0.053	-0.131	0.024	0.1753	0.1753
Mean cortical thickness	non-APOE PGLS	0.005	-0.071	0.082	0.8895	0.8895
Mean FA values	non-APOE PGLS	-0.009	-0.088	0.070	0.8246	0.9610
Total SVD score	non-APOE PGLS	0.830	0.647	1.060	0.1373	0.1373
SVD score (without WMHs)	non-APOE PGLS	0.829	0.611	1.118	0.2230	0.2230

PGLS = polygenic longevity score, OR = Odd ratio, SVD = small vessel disease, LCI = lower confidence limit, UCI = upper confidence limit, and P = p-value.

sTable 4: Primary results - Associations between the PGLSs and raw SVD score (Ordinal regression)

Variable	OR	LCI	UCI	P	Adjusted P
**SVD score (0-4)**					
APOE PGLS	0.869	0.742	1.015	0.0772	0.4460
non-APOE PGLS	0.874	0.747	1.021	0.0906	0.4488

PGLS = polygenic longevity score, OR = Odd ratio, SVD = small vessel disease, LCI = lower confidence limit, UCI = upper confidence limit, and P = p-value.

sTable 5: Primary results - Interaction between sex and PGLSs in relation to global brain MRI measures

Brain Measure	Interaction Term	Beta/OR	LCI	UCI	P-value
Total GM volume	APOE PGLS*Female	-0.083	-0.233	0.067	0.2775
Subcortical GM volume	APOE PGLS*Female	-0.124	-0.254	0.006	0.0611
Total WM volume	APOE PGLS*Female	-0.189	-0.334	-0.043	**0.0110**
Total WMHs volume	APOE PGLS*Female	0.234	0.084	0.384	**0.0023**
Log Total WMHs volume	APOE PGLS*Female	0.238	0.088	0.388	**0.0019**
Mean cortical thickness	APOE PGLS*Female	-0.095	-0.245	0.055	0.2161
Mean FA values	APOE PGLS*Female	-0.093	-0.246	0.060	0.2309
Total SVD score	APOE PGLS*Female	1.728	1.066	2.814	**0.0269**
SVD score (without WMHs)	APOE PGLS*Female	1.751	0.970	3.176	0.0636
Total GM volume	non-APOE PGLS*Female	-0.101	-0.250	0.049	0.1855
Subcortical GM volume	non-APOE PGLS*Female	-0.123	-0.253	0.006	0.0619
Total WM volume	non-APOE PGLS*Female	-0.173	-0.318	-0.027	**0.0200**
Total WMHs volume	non-APOE PGLS*Female	0.207	0.058	0.357	**0.0068**
Log Total WMHs volume	non-APOE PGLS*Female	0.200	0.050	0.350	**0.0090**
Mean cortical thickness	non-APOE PGLS*Female	-0.101	-0.251	0.048	0.1843
Mean FA values	non-APOE PGLS*Female	-0.080	-0.233	0.073	0.3064
Total SVD score	non-APOE PGLS*Female	1.772	1.092	2.889	**0.0209**
SVD score (without WMHs)	non-APOE PGLS*Female	1.965	1.088	3.570	**0.0256**

PGLS = polygenic longevity score, OR = Odd ratio, SVD = small vessel disease, LCI = lower confidence limit, UCI = upper confidence limit, and P = p-value.

sTable 6: Primary results - Sex-stratified associations between PGLSs and global MRI measures

Group	Brain measures	PGLSs	Beta/OR	LCI	UCI	P	Adjusted P
Males	Total WM volume	APOE PGLS	0.122	-0.005	0.250	0.0596	0.0596
Males	Total WMHs volume	APOE PGLS	-0.190	-0.326	-0.054	0.0062	**0.0185**
Males	Log Total WMHs volume	APOE PGLS	-0.176	-0.317	-0.035	0.0144	**0.0216**
Females	Total WM volume	APOE PGLS	-0.047	-0.135	0.041	0.2911	0.3605
Females	Total WMHs volume	APOE PGLS	0.040	-0.046	0.126	0.3605	0.3605
Females	Log Total WMHs volume	APOE PGLS	0.048	-0.032	0.128	0.2381	0.3605
Males	Total SVD score	APOE PGLS	0.632	0.449	0.880	0.0074	**0.0143**
Males	SVD score (without WMHs)	APOE PGLS	0.593	0.386	0.895	0.0143	**0.0143**
Females	Total SVD score	APOE PGLS	1.117	0.768	1.614	0.5564	0.6764
Females	SVD score (without WMHs)	APOE PGLS	1.100	0.697	1.718	0.6764	0.6764
Males	Total WM volume	non-APOE PGLS	0.098	-0.029	0.225	0.1292	0.1292
Males	Total WMHs volume	non-APOE PGLS	-0.170	-0.306	-0.035	0.0139	**0.0418**
Males	Log Total WMHs volume	non-APOE PGLS	-0.149	-0.290	-0.009	0.0376	0.0564
Females	Total WM volume	non-APOE PGLS	-0.054	-0.141	0.034	0.2274	0.4988
Females	Total WMHs volume	non-APOE PGLS	0.030	-0.056	0.115	0.4988	0.4988
Females	Log Total WMHs volume	non-APOE PGLS	0.032	-0.047	0.112	0.4230	0.4988
Males	Total SVD score	non-APOE PGLS	0.626	0.443	0.873	0.0065	**0.0120**
Males	SVD score (without WMHs)	non-APOE PGLS	0.582	0.377	0.881	0.0120	**0.0120**
Females	Total SVD score	non-APOE PGLS	1.129	0.779	1.626	0.5143	0.5143
Females	SVD score (without WMHs)	non-APOE PGLS	1.202	0.768	1.865	0.4140	0.5143

PGLS = polygenic longevity score, OR = Odd ratio, SVD = small vessel disease, LCI = lower confidence limit, UCI = upper confidence limit, and P = p-value.

sTable 7: Primary results - Association between PGLSs and vascular factors (WMH and SVD score) adjusted for possible confounders.

Phenotype	PGLSs	Beta/OR	LCI	UCI	SE	P	Adjusted P
Total WMHs volume	APOE PGLS	-0.062	-0.139	0.015	0.039	0.1132	0.1132
Log Total WMHs volume	APOE PGLS	-0.057	-0.134	0.021	0.039	0.1523	0.1523
Total SVD score	APOE PGLS	0.869	0.672	1.119	0.130	0.2777	0.2777
SVD score (without WMHs)	APOE PGLS	0.807	0.586	1.103	0.161	0.1823	0.1823
Total WMHs volume	non-APOE PGLS	-0.062	-0.138	0.015	0.039	0.1138	0.1138
Log Total WMHs volume	non-APOE PGLS	-0.054	-0.131	0.023	0.039	0.1692	0.1692
Total SVD score	non-APOE PGLS	0.858	0.662	1.106	0.131	0.2403	0.2403
SVD score (without WMHs)	non-APOE PGLS	0.826	0.599	1.130	0.162	0.2371	0.2371

PGLS = polygenic longevity score, OR = Odd ratio, SVD = small vessel disease, LCI = lower confidence limit, UCI = upper confidence limit, and P = p-value.

sTable 8: Primary results - Interaction between sex and PGLSs in relation to vascular factors

Brain Measure	Interaction Term	Beta/OR	LCI	UCI	P-value
Total WMHs volume	APOE PGLS*Female	0.206	0.059	0.354	**0.0062**
Log Total WMHs volume	APOE PGLS*Female	0.218	0.069	0.367	**0.0043**
Total SVD score	APOE PGLS*Female	1.615	0.991	2.644	0.0548
SVD score (without WMHs)	APOE PGLS*Female	1.665	0.916	3.036	0.0946
Total WMHs volume	non-APOE PGLS*Female	0.183	0.036	0.331	**0.0148**
Log Total WMHs volume	non-APOE PGLS*Female	0.184	0.035	0.333	**0.0155**
Total SVD score	non-APOE PGLS*Female	1.669	1.021	2.742	**0.0418**
SVD score (without WMHs)	non-APOE PGLS*Female	1.881	1.031	3.451	**0.0399**

PGLS = polygenic longevity score, OR = Odd ratio, SVD = small vessel disease, LCI = lower confidence limit, UCI = upper confidence limit, and P = p-value. Vascular factors: years of education, smoking, hypertension, BMI, diabetes, and hypercholesterolemia.

sTable 9: Primary results - Sex-stratified associations between PGLSs and vascular factors adjusted for possible confounders

Group	Brain measures	PGLSs	Beta/OR	LCI	UCI	P	Adjusted P
Males	Total WMHs volume	APOE PGLS	-0.061	-0.139	0.015	0.1193	0.1193
Females	Total WMHs volume	APOE PGLS	-0.061	-0.139	0.015	0.1193	0.1193
Males	Log Total WMHs volume	APOE PGLS	-0.057	-0.134	0.021	0.1465	0.1465
Females	Log Total WMHs volume	APOE PGLS	-0.057	-0.134	0.021	0.1465	0.1465
Males	Total SVD score	APOE PGLS	0.698	0.482	0.999	0.0523	0.0523

Females	Total SVD score	APOE PGLS	1.075	0.733	1.571	0.7068	0.7068
Males	SVD score (without WMHs)	APOE PGLS	0.624	0.388	0.982	0.0454	0.0454
Females	SVD score (without WMHs)	APOE PGLS	1.037	0.642	1.662	0.8792	0.8792
Males	Total WMHs volume	non-APOE PGLS	-0.061	-0.138	0.015	0.1188	0.1188
Females	Total WMHs volume	non-APOE PGLS	-0.061	-0.138	0.015	0.1188	0.1188
Males	Log Total WMHs volume	non-APOE PGLS	-0.055	-0.131	0.023	0.1641	0.1641
Females	Log Total WMHs volume	non-APOE PGLS	-0.055	-0.131	0.023	0.1641	0.1641
Males	Total SVD score	non-APOE PGLS	0.674	0.464	0.968	0.0352	**0.0352**
Females	Total SVD score	non-APOE PGLS	1.079	0.736	1.575	0.6923	0.6923
Males	SVD score (without WMHs)	non-APOE PGLS	0.591	0.363	0.936	0.0284	**0.0284**
Females	SVD score (without WMHs)	non-APOE PGLS	1.134	0.708	1.808	0.5962	0.5962

PGLS = polygenic longevity score, OR = Odd ratio, SVD = small vessel disease, LCI = lower confidence limit, UCI = upper confidence limit, and P = p-value.

sTable 10: Primary results - Interaction between sex and PGLSs in relation to raw SVD score ranging from 0 to 4 (Ordinal regression)

Variable	OR	LCI	UCI	P
SVD score (0-4)				
APOE PGLS*Female	1.115	0.824	1.510	0.4817
non-APOE PGLS*Female	1.134	0.839	1.535	0.4137

PGLS = polygenic longevity score, OR = Odd ratio, SVD = small vessel disease, LCI = lower confidence limit, UCI = upper confidence limit, and P = p-value.

sTable 11: Secondary results - Association between PGLSs and subcortical volumes in the total sample

Phenotype	PGLSs	Beta	LCI	UCI	P	Adjusted P
Hippocampal volume	APOE PGLS	0.007	-0.070	0.084	0.8588	0.8588
Amygdala volume	APOE PGLS	0.037	-0.041	0.114	0.3542	0.6972
Caudate volume	APOE PGLS	-0.027	-0.105	0.051	0.4980	0.6972
Thalamus volume	APOE PGLS	0.041	-0.037	0.119	0.3015	0.6972
Putamen volume	APOE PGLS	0.032	-0.045	0.110	0.4105	0.6972
Accumbens volume	APOE PGLS	-0.014	-0.092	0.064	0.7288	0.8502
Pallidum volume	APOE PGLS	0.040	-0.038	0.117	0.3148	0.6972
Hippocampal volume	non-APOE PGLS	0.011	-0.066	0.088	0.7833	0.7833
Amygdala volume	non-APOE PGLS	0.039	-0.039	0.116	0.3268	0.6214
Caudate volume	non-APOE PGLS	-0.022	-0.099	0.056	0.5832	0.6805
Thalamus volume	non-APOE PGLS	0.037	-0.041	0.114	0.3551	0.6214
Putamen volume	non-APOE PGLS	0.041	-0.036	0.118	0.2915	0.6214
Accumbens volume	non-APOE PGLS	-0.023	-0.100	0.055	0.5656	0.6805
Pallidum volume	non-APOE PGLS	0.042	-0.036	0.119	0.2911	0.6214

PGLS = polygenic longevity score, LCI = lower confidence limit, UCI = upper confidence limit, and P = p-value.

sTable 12: Secondary results - Association between PGLSs and regional cortical thicknesses in the total sample

Brain measures	PGLSs	Beta	LCI	UCI	P	Adjusted P
bankssts thickness	APOE PGLS	-0.022	-0.099	0.055	0.5675	0.9992
caudalanteriorcingulate thickness	APOE PGLS	-0.033	-0.109	0.044	0.4017	0.9992
caudalmiddlefrontal thickness	APOE PGLS	0.014	-0.063	0.092	0.7163	0.9992
cuneus thickness	APOE PGLS	-0.038	-0.115	0.040	0.3399	0.9992
entorhinal thickness	APOE PGLS	-0.050	-0.127	0.027	0.2059	0.9992

fusiform thickness	APOE PGLS	-0.007	-0.084	0.070	0.8562	0.9992
inferiorparietal thickness	APOE PGLS	0.049	-0.029	0.126	0.2187	0.9992
inferiortemporal thickness	APOE PGLS	-0.019	-0.096	0.059	0.6388	0.9992
isthmuscingulate thickness	APOE PGLS	0.001	-0.077	0.078	0.9819	0.9992
lateraloccipital thickness	APOE PGLS	-0.006	-0.083	0.072	0.8890	0.9992
lateralorbitofrontal thickness	APOE PGLS	-0.063	-0.141	0.014	0.1093	0.9992
lingual thickness	APOE PGLS	0.026	-0.052	0.103	0.5137	0.9992
medialorbitofrontal thickness	APOE PGLS	0.006	-0.072	0.084	0.8792	0.9992
middletemporal thickness	APOE PGLS	-0.022	-0.099	0.055	0.5753	0.9992
parahippocampal thickness	APOE PGLS	-0.012	-0.087	0.063	0.7570	0.9992
paracentral thickness	APOE PGLS	0.023	-0.055	0.101	0.5651	0.9992
parsopercularis thickness	APOE PGLS	0.040	-0.037	0.118	0.3052	0.9992
parsorbitalis thickness	APOE PGLS	-0.025	-0.101	0.051	0.5183	0.9992
parstriangularis thickness	APOE PGLS	0.070	-0.007	0.147	0.0732	0.9992
pericalcarine thickness	APOE PGLS	0.008	-0.070	0.086	0.8433	0.9992
postcentral thickness	APOE PGLS	-0.012	-0.089	0.064	0.7494	0.9992
posteriorcingulate thickness	APOE PGLS	0.036	-0.041	0.114	0.3608	0.9992
precentral thickness	APOE PGLS	-0.016	-0.093	0.062	0.6930	0.9992
precuneus thickness	APOE PGLS	-0.020	-0.098	0.057	0.6091	0.9992
rostralanteriorcingulate thickness	APOE PGLS	0.000	-0.076	0.077	0.9926	0.9992
rostralmiddlefrontal thickness	APOE PGLS	0.024	-0.054	0.101	0.5483	0.9992
superiorfrontal thickness	APOE PGLS	0.045	-0.032	0.121	0.2502	0.9992
superiorparietal thickness	APOE PGLS	0.001	-0.076	0.078	0.9778	0.9992
superiortemporal thickness	APOE PGLS	-0.015	-0.092	0.063	0.7101	0.9992
supramarginal thickness	APOE PGLS	0.016	-0.061	0.093	0.6789	0.9992
frontalpole thickness	APOE PGLS	-0.014	-0.090	0.062	0.7179	0.9992
temporalpole thickness	APOE PGLS	0.005	-0.072	0.083	0.8929	0.9992
transversetemporal thickness	APOE PGLS	0.017	-0.061	0.094	0.6758	0.9992
insula thickness	APOE PGLS	0.033	-0.045	0.110	0.4064	0.9992
bankssts thickness	non-APOE PGLS	-0.018	-0.094	0.059	0.6499	0.9992
caudalanteriorcingulate thickness	non-APOE PGLS	-0.043	-0.120	0.033	0.2651	0.9992
caudalmiddlefrontal thickness	non-APOE PGLS	0.006	-0.072	0.083	0.8866	0.9992
cuneus thickness	non-APOE PGLS	-0.037	-0.114	0.041	0.3511	0.9992
entorhinal thickness	non-APOE PGLS	-0.048	-0.125	0.028	0.2179	0.9992
fusiform thickness	non-APOE PGLS	-0.003	-0.080	0.074	0.9390	0.9992
inferiorparietal thickness	non-APOE PGLS	0.049	-0.028	0.126	0.2147	0.9992
inferiortemporal thickness	non-APOE PGLS	-0.013	-0.091	0.064	0.7344	0.9992
isthmuscingulate thickness	non-APOE PGLS	0.002	-0.075	0.080	0.9544	0.9992
lateraloccipital thickness	non-APOE PGLS	-0.008	-0.086	0.069	0.8305	0.9992
lateralorbitofrontal thickness	non-APOE PGLS	-0.063	-0.140	0.014	0.1110	0.9992
lingual thickness	non-APOE PGLS	0.027	-0.050	0.105	0.4855	0.9992
medialorbitofrontal thickness	non-APOE PGLS	0.013	-0.065	0.090	0.7481	0.9992
middletemporal thickness	non-APOE PGLS	-0.030	-0.107	0.047	0.4419	0.9992
parahippocampal thickness	non-APOE PGLS	-0.016	-0.091	0.059	0.6682	0.9992
paracentral thickness	non-APOE PGLS	0.013	-0.064	0.091	0.7361	0.9992
parsopercularis thickness	non-APOE PGLS	0.036	-0.042	0.113	0.3645	0.9992

parsorbitalis thickness	non-APOE PGLS	-0.023	-0.099	0.052	0.5469	0.9992
parstriangularis thickness	non-APOE PGLS	0.075	-0.002	0.151	0.0565	0.9992
pericalcarine thickness	non-APOE PGLS	0.009	-0.068	0.086	0.8212	0.9992
postcentral thickness	non-APOE PGLS	-0.023	-0.099	0.053	0.5515	0.9992
posteriorcingulate thickness	non-APOE PGLS	0.034	-0.043	0.111	0.3838	0.9992
precentral thickness	non-APOE PGLS	-0.020	-0.097	0.057	0.6049	0.9992
precuneus thickness	non-APOE PGLS	-0.029	-0.107	0.048	0.4533	0.9992
rostralanteriorcingulate thickness	non-APOE PGLS	0.000	-0.076	0.076	0.9992	0.9992
rostralmiddlefrontal thickness	non-APOE PGLS	0.027	-0.049	0.104	0.4837	0.9992
superiorfrontal thickness	non-APOE PGLS	0.042	-0.034	0.119	0.2748	0.9992
superiorparietal thickness	non-APOE PGLS	-0.002	-0.079	0.074	0.9500	0.9992
superiortemporal thickness	non-APOE PGLS	-0.016	-0.093	0.061	0.6921	0.9992
supramarginal thickness	non-APOE PGLS	0.014	-0.063	0.090	0.7228	0.9992
frontalpole thickness	non-APOE PGLS	-0.012	-0.087	0.064	0.7627	0.9992
temporalpole thickness	non-APOE PGLS	0.010	-0.067	0.087	0.7955	0.9992
transversetemporal thickness	non-APOE PGLS	0.002	-0.075	0.079	0.9619	0.9992
insula thickness	non-APOE PGLS	0.034	-0.044	0.111	0.3948	0.9992

PGLS = polygenic longevity score, LCI = lower confidence limit, UCI = upper confidence limit, and P = p-value.

sTable 13: Secondary results - Association between PGLSs and FA WM fibre tracts in the total sample

Brain measures	PGLSs	Beta	LCI	UCI	P	Adjusted P
FA body of corpus callosum	APOE PGLS	-0.008	-0.087	0.070	0.8328	0.9909
FA fornix	APOE PGLS	0.018	-0.059	0.094	0.6467	0.9909
FA genu of corpus callosum	APOE PGLS	0.002	-0.077	0.082	0.9516	0.9909
FA middle cerebellar peduncle	APOE PGLS	0.000	-0.078	0.079	0.9907	0.9909
FA pontine crossing tract	APOE PGLS	0.065	-0.015	0.144	0.1103	0.9909
FA splenium of corpus callosum	APOE PGLS	0.017	-0.062	0.096	0.6700	0.9909
FA anterior corona radiata	APOE PGLS	0.002	-0.077	0.082	0.9539	0.9909
FA anterior limb of internal capsule	APOE PGLS	0.015	-0.064	0.094	0.7152	0.9909
FA cerebral peduncle	APOE PGLS	-0.004	-0.081	0.074	0.9264	0.9909
FA cingulum hippocampus	APOE PGLS	-0.007	-0.085	0.071	0.8613	0.9909
FA cingulum	APOE PGLS	0.001	-0.078	0.080	0.9839	0.9909
FA corticospinal tract	APOE PGLS	-0.027	-0.106	0.052	0.4990	0.9909
FA external capsule	APOE PGLS	-0.033	-0.112	0.046	0.4074	0.9909
FA fornix cres	APOE PGLS	0.026	-0.053	0.105	0.5187	0.9909
FA inferior cerebellar peduncle	APOE PGLS	-0.057	-0.135	0.021	0.1544	0.9909
FA medial lemniscus	APOE PGLS	-0.015	-0.093	0.064	0.7109	0.9909
FA posterior corona radiata	APOE PGLS	0.041	-0.038	0.119	0.3115	0.9909
FA posterior limb of internal capsule	APOE PGLS	-0.024	-0.101	0.052	0.5325	0.9909
FA posterior thalamic radiation	APOE PGLS	0.059	-0.020	0.138	0.1403	0.9909
FA retrolenticular part of internal capsule	APOE PGLS	-0.007	-0.085	0.070	0.8534	0.9909
FA sagittal stratum	APOE PGLS	0.017	-0.061	0.096	0.6642	0.9909
FA superior cerebellar peduncle	APOE PGLS	-0.012	-0.091	0.067	0.7664	0.9909
FA superior corona radiata	APOE PGLS	0.021	-0.058	0.099	0.6048	0.9909
FA superior fronto.occipital fasciculus	APOE PGLS	0.008	-0.072	0.087	0.8521	0.9909
FA superior longitudinal fasciculus	APOE PGLS	-0.004	-0.083	0.075	0.9160	0.9909

FA tapetum	APOE PGLS	-0.002	-0.081	0.077	0.9590	0.9909
FA uncinate fasciculus	APOE PGLS	-0.046	-0.124	0.033	0.2543	0.9909
FA body of corpus callosum	non-APOE PGLS	-0.016	-0.095	0.062	0.6842	0.9909
FA fornix	non-APOE PGLS	0.016	-0.061	0.092	0.6864	0.9909
FA genu of corpus callosum	non-APOE PGLS	-0.013	-0.092	0.066	0.7480	0.9909
FA middle cerebellar peduncle	non-APOE PGLS	-0.021	-0.100	0.057	0.5921	0.9909
FA pontine crossing tract	non-APOE PGLS	0.052	-0.027	0.132	0.1955	0.9909
FA splenium of corpus callosum	non-APOE PGLS	0.000	-0.079	0.080	0.9903	0.9909
FA anterior corona radiata	non-APOE PGLS	0.000	-0.080	0.079	0.9909	0.9909
FA anterior limb of internal capsule	non-APOE PGLS	0.009	-0.070	0.088	0.8191	0.9909
FA cerebral peduncle	non-APOE PGLS	-0.015	-0.092	0.063	0.7105	0.9909
FA cingulum hippocampus	non-APOE PGLS	-0.016	-0.095	0.062	0.6798	0.9909
FA cingulum	non-APOE PGLS	-0.011	-0.090	0.068	0.7823	0.9909
FA corticospinal tract	non-APOE PGLS	-0.030	-0.109	0.049	0.4562	0.9909
FA external capsule	non-APOE PGLS	-0.044	-0.123	0.035	0.2761	0.9909
FA fornix cres	non-APOE PGLS	0.019	-0.060	0.098	0.6392	0.9909
FA inferior cerebellar peduncle	non-APOE PGLS	-0.070	-0.148	0.008	0.0788	0.9909
FA medial lemniscus	non-APOE PGLS	-0.017	-0.096	0.061	0.6669	0.9909
FA posterior corona radiata	non-APOE PGLS	0.039	-0.039	0.118	0.3276	0.9909
FA posterior limb of internal capsule	non-APOE PGLS	-0.038	-0.115	0.038	0.3242	0.9909
FA posterior thalamic radiation	non-APOE PGLS	0.049	-0.030	0.128	0.2226	0.9909
FA retrolenticular part of internal capsule	non-APOE PGLS	-0.018	-0.096	0.060	0.6538	0.9909
FA sagittal stratum	non-APOE PGLS	0.011	-0.068	0.090	0.7850	0.9909
FA superior cerebellar peduncle	non-APOE PGLS	-0.018	-0.097	0.060	0.6468	0.9909
FA superior corona radiata	non-APOE PGLS	0.013	-0.066	0.091	0.7550	0.9909
FA superior fronto.occipital fasciculus	non-APOE PGLS	0.002	-0.077	0.081	0.9545	0.9909
FA superior longitudinal fasciculus	non-APOE PGLS	-0.016	-0.096	0.063	0.6824	0.9909
FA tapetum	non-APOE PGLS	-0.014	-0.093	0.065	0.7220	0.9909
FA uncinate fasciculus	non-APOE PGLS	-0.049	-0.128	0.029	0.2184	0.9909

PGLS = polygenic longevity score, FA = fractional anisotropy, LCI = lower confidence limit, UCI = upper confidence limit, and P = p-value.

sTable 14: Secondary results - Interaction between sex and PGLSs in relation to subcortical brain volumes

Brain Measure	Interaction Term	Beta	LCI	UCI	P-value
Hippocampal volume	APOE PGLS*Female	0.028	-0.123	0.178	0.7168
Amygdala volume	APOE PGLS*Female	0.048	-0.103	0.198	0.5364
Thalamus volume	APOE PGLS*Female	-0.118	-0.269	0.034	0.1278
Caudate volume	APOE PGLS*Female	-0.095	-0.246	0.056	0.2174
Putamen volume	APOE PGLS*Female	-0.055	-0.206	0.095	0.4695
Accumbens volume	APOE PGLS*Female	-0.144	-0.296	0.007	0.0614

Pallidum volume	APOE PGLS*Female	-0.166	-0.317	-0.015	**0.0312**
Hippocampal volume	non-APOE PGLS*Female	0.021	-0.129	0.171	0.7862
Amygdala volume	non-APOE PGLS*Female	0.032	-0.119	0.182	0.6802
Thalamus volume	non-APOE PGLS*Female	-0.130	-0.281	0.021	0.0925
Caudate volume	non-APOE PGLS*Female	-0.107	-0.258	0.044	0.1645
Putamen volume	non-APOE PGLS*Female	-0.053	-0.203	0.097	0.4841
Accumbens volume	non-APOE PGLS*Female	-0.150	-0.301	0.001	0.0515
Pallidum volume	non-APOE PGLS*Female	-0.170	-0.321	-0.020	**0.0263**

PGLS = polygenic longevity score, LCI = lower confidence limit, UCI = upper confidence limit, and P = p-value.

sTable 15: Secondary results - Interaction between sex and PGLSs in relation to regional cortical thickness

Brain Measure	Interaction Term	Beta	LCI	UCI	P-value
bankssts thickness	APOE PGLS*Female	-0.052	-0.202	0.098	0.4939
caudalanteriorcingulate thickness	APOE PGLS*Female	-0.013	-0.162	0.136	0.8646
caudalmiddlefrontal thickness	APOE PGLS*Female	-0.077	-0.228	0.074	0.3179
cuneus thickness	APOE PGLS*Female	-0.057	-0.208	0.094	0.4596
entorhinal thickness	APOE PGLS*Female	-0.051	-0.201	0.098	0.5007
fusiform thickness	APOE PGLS*Female	0.029	-0.121	0.179	0.7051
inferiorparietal thickness	APOE PGLS*Female	0.056	-0.095	0.207	0.4669
inferiortemporal thickness	APOE PGLS*Female	-0.056	-0.207	0.095	0.4653
isthmuscingulate thickness	APOE PGLS*Female	-0.014	-0.165	0.137	0.8555
lateraloccipital thickness	APOE PGLS*Female	0.010	-0.141	0.161	0.8955
lateralorbitofrontal thickness	APOE PGLS*Female	0.055	-0.096	0.205	0.4773
lingual thickness	APOE PGLS*Female	-0.054	-0.205	0.097	0.4851
medialorbitofrontal thickness	APOE PGLS*Female	-0.101	-0.252	0.051	0.1920
middletemporal thickness	APOE PGLS*Female	0.026	-0.124	0.177	0.7299
parahippocampal thickness	APOE PGLS*Female	0.065	-0.082	0.211	0.3873
paracentral thickness	APOE PGLS*Female	-0.097	-0.248	0.055	0.2106
parsopercularis thickness	APOE PGLS*Female	-0.187	-0.337	-0.036	**0.0150**
parsorbitalis thickness	APOE PGLS*Female	-0.108	-0.256	0.039	0.1507
parstriangularis thickness	APOE PGLS*Female	-0.192	-0.341	-0.042	**0.0120**
pericalcarine thickness	APOE PGLS*Female	-0.075	-0.226	0.077	0.3333
postcentral thickness	APOE PGLS*Female	-0.081	-0.230	0.067	0.2832
posteriorcingulate thickness	APOE PGLS*Female	-0.016	-0.167	0.135	0.8319
precentral thickness	APOE PGLS*Female	-0.113	-0.263	0.037	0.1394
precuneus thickness	APOE PGLS*Female	-0.062	-0.213	0.089	0.4214
rostralanteriorcingulate thickness	APOE PGLS*Female	-0.079	-0.228	0.069	0.2961
rostralmiddlefrontal thickness	APOE PGLS*Female	-0.086	-0.236	0.064	0.2614
superiorfrontal thickness	APOE PGLS*Female	-0.024	-0.173	0.125	0.7538
superiorparietal thickness	APOE PGLS*Female	-0.017	-0.167	0.134	0.8283
superiortemporal thickness	APOE PGLS*Female	-0.082	-0.233	0.068	0.2827
supramarginal thickness	APOE PGLS*Female	-0.144	-0.293	0.006	0.0594
frontalpole thickness	APOE PGLS*Female	-0.016	-0.164	0.131	0.8269
temporalpole thickness	APOE PGLS*Female	-0.099	-0.250	0.051	0.1958
transversetemporal thickness	APOE PGLS*Female	0.022	-0.129	0.173	0.7732

insula thickness	APOE PGLS*Female	-0.198	-0.349	-0.048	**0.0098**
bankssts thickness	non-APOE PGLS*Female	-0.072	-0.221	0.078	0.3463
caudalanteriorcingulate thickness	non-APOE PGLS*Female	-0.002	-0.150	0.147	0.9823
caudalmiddlefrontal thickness	non-APOE PGLS*Female	-0.076	-0.227	0.074	0.3203
cuneus thickness	non-APOE PGLS*Female	-0.061	-0.212	0.090	0.4265
entorhinal thickness	non-APOE PGLS*Female	-0.021	-0.170	0.128	0.7831
fusiform thickness	non-APOE PGLS*Female	0.013	-0.137	0.162	0.8685
inferiorparietal thickness	non-APOE PGLS*Female	0.043	-0.107	0.193	0.5738
inferiortemporal thickness	non-APOE PGLS*Female	-0.063	-0.214	0.088	0.4114
isthmuscingulate thickness	non-APOE PGLS*Female	-0.003	-0.154	0.147	0.9662
lateraloccipital thickness	non-APOE PGLS*Female	-0.007	-0.157	0.144	0.9301
lateralorbitofrontal thickness	non-APOE PGLS*Female	0.043	-0.108	0.193	0.5777
lingual thickness	non-APOE PGLS*Female	-0.067	-0.218	0.083	0.3790
medialorbitofrontal thickness	non-APOE PGLS*Female	-0.113	-0.264	0.038	0.1407
middletemporal thickness	non-APOE PGLS*Female	0.011	-0.139	0.161	0.8894
parahippocampal thickness	non-APOE PGLS*Female	0.047	-0.099	0.193	0.5306
paracentral thickness	non-APOE PGLS*Female	-0.097	-0.248	0.054	0.2062
parsopercularis thickness	non-APOE PGLS*Female	-0.189	-0.338	-0.039	**0.0137**
parsorbitalis thickness	non-APOE PGLS*Female	-0.127	-0.274	0.020	0.0908
parstriangularis thickness	non-APOE PGLS*Female	-0.207	-0.356	-0.059	**0.0064**
pericalcarine thickness	non-APOE PGLS*Female	-0.087	-0.238	0.064	0.2580
postcentral thickness	non-APOE PGLS*Female	-0.090	-0.238	0.057	0.2303
posteriorcingulate thickness	non-APOE PGLS*Female	0.006	-0.144	0.157	0.9359
precentral thickness	non-APOE PGLS*Female	-0.115	-0.265	0.034	0.1305
precuneus thickness	non-APOE PGLS*Female	-0.052	-0.203	0.098	0.4946
rostralanteriorcingulate thickness	non-APOE PGLS*Female	-0.088	-0.236	0.060	0.2443
rostralmiddlefrontal thickness	non-APOE PGLS*Female	-0.091	-0.241	0.059	0.2332
superiorfrontal thickness	non-APOE PGLS*Female	-0.017	-0.166	0.131	0.8202
superiorparietal thickness	non-APOE PGLS*Female	-0.005	-0.155	0.144	0.9428
superiortemporal thickness	non-APOE PGLS*Female	-0.104	-0.254	0.046	0.1723
supramarginal thickness	non-APOE PGLS*Female	-0.160	-0.309	-0.011	**0.0350**
frontalpole thickness	non-APOE PGLS*Female	-0.021	-0.168	0.127	0.7832
temporalpole thickness	non-APOE PGLS*Female	-0.092	-0.242	0.058	0.2305
transversetemporal thickness	non-APOE PGLS*Female	0.010	-0.141	0.160	0.9001
insula thickness	non-APOE PGLS*Female	-0.201	-0.351	-0.051	**0.0085**

PGLS = polygenic longevity score, LCI = lower confidence limit, UCI = upper confidence limit, and P = p-value.

sTable 16: Secondary results - Interaction between sex and PGLSs in relation to FA WM fibre tracts

Brain Measure	Interaction Term	Beta	LCI	UCI	P-value
FA body of corpus callosum	APOE PGLS*Female	-0.151	-0.304	0.001	0.0518
FA fornix	APOE PGLS*Female	-0.028	-0.176	0.121	0.7156
FA genu of corpus callosum	APOE PGLS*Female	-0.059	-0.214	0.095	0.4494
FA middle cerebellar peduncle	APOE PGLS*Female	-0.107	-0.260	0.045	0.1679
FA pontine crossing tract	APOE PGLS*Female	-0.098	-0.252	0.056	0.2127
FA splenium of corpus callosum	APOE PGLS*Female	-0.089	-0.243	0.064	0.2541

FA anterior corona radiata	APOE PGLS*Female	-0.064	-0.218	0.089	0.4116
FA anterior limb of internal capsule	APOE PGLS*Female	-0.030	-0.183	0.123	0.6999
FA cerebral peduncle	APOE PGLS*Female	0.032	-0.119	0.182	0.6809
FA cingulum hippocampus	APOE PGLS*Female	-0.062	-0.214	0.090	0.4206
FA cingulum	APOE PGLS*Female	-0.137	-0.290	0.016	0.0797
FA corticospinal tract	APOE PGLS*Female	-0.105	-0.258	0.048	0.1771
FA external capsule	APOE PGLS*Female	-0.117	-0.271	0.036	0.1330
FA fornix cres	APOE PGLS*Female	-0.152	-0.306	0.002	0.0526
FA inferior cerebellar peduncle	APOE PGLS*Female	-0.067	-0.218	0.085	0.3891
FA medial lemniscus	APOE PGLS*Female	-0.096	-0.249	0.056	0.2150
FA posterior corona radiata	APOE PGLS*Female	-0.082	-0.235	0.071	0.2908
FA posterior limb of internal capsule	APOE PGLS*Female	0.046	-0.102	0.194	0.5425
FA posterior thalamic radiation	APOE PGLS*Female	-0.128	-0.281	0.025	0.1002
FA retrolenticular part of internal capsule	APOE PGLS*Female	-0.050	-0.201	0.101	0.5177
FA sagittal stratum	APOE PGLS*Female	-0.133	-0.286	0.020	0.0887
FA superior cerebellar peduncle	APOE PGLS*Female	-0.119	-0.272	0.034	0.1260
FA superior corona radiata	APOE PGLS*Female	0.034	-0.119	0.187	0.6637
FA superior fronto.occipital fasciculus	APOE PGLS*Female	-0.075	-0.229	0.078	0.3371
FA superior longitudinal fasciculus	APOE PGLS*Female	-0.116	-0.269	0.038	0.1393
FA tapetum	APOE PGLS*Female	-0.064	-0.217	0.090	0.4140
FA uncinate fasciculus	APOE PGLS*Female	0.053	-0.099	0.206	0.4913
FA body of corpus callosum	non-APOE PGLS*Female	-0.136	-0.289	0.017	0.0817
FA fornix	non-APOE PGLS*Female	-0.028	-0.177	0.120	0.7090
FA genu of corpus callosum	non-APOE PGLS*Female	-0.056	-0.211	0.098	0.4725
FA middle cerebellar peduncle	non-APOE PGLS*Female	-0.096	-0.249	0.057	0.2194
FA pontine crossing tract	non-APOE PGLS*Female	-0.073	-0.228	0.081	0.3509
FA splenium of corpus callosum	non-APOE PGLS*Female	-0.066	-0.220	0.088	0.4016
FA anterior corona radiata	non-APOE PGLS*Female	-0.067	-0.221	0.087	0.3908
FA anterior limb of internal capsule	non-APOE PGLS*Female	-0.021	-0.175	0.132	0.7836
FA cerebral peduncle	non-APOE PGLS*Female	0.031	-0.120	0.182	0.6881
FA cingulum hippocampus	non-APOE PGLS*Female	-0.048	-0.200	0.104	0.5353
FA cingulum	non-APOE PGLS*Female	-0.127	-0.280	0.026	0.1044
FA corticospinal tract	non-APOE PGLS*Female	-0.099	-0.252	0.054	0.2043
FA external capsule	non-APOE PGLS*Female	-0.102	-0.255	0.052	0.1927
FA fornix cres	non-APOE PGLS*Female	-0.144	-0.298	0.010	0.0659
FA inferior cerebellar peduncle	non-APOE PGLS*Female	-0.055	-0.207	0.097	0.4753
FA medial lemniscus	non-APOE PGLS*Female	-0.099	-0.252	0.054	0.2038
FA posterior corona radiata	non-APOE PGLS*Female	-0.065	-0.218	0.089	0.4078
FA posterior limb of internal capsule	non-APOE PGLS*Female	0.061	-0.088	0.209	0.4229
FA posterior thalamic radiation	non-APOE PGLS*Female	-0.119	-0.272	0.034	0.1266
FA retrolenticular part of internal capsule	non-APOE PGLS*Female	-0.039	-0.190	0.112	0.6128
FA sagittal stratum	non-APOE PGLS*Female	-0.118	-0.271	0.036	0.1320
FA superior cerebellar peduncle	non-APOE PGLS*Female	-0.108	-0.261	0.045	0.1655
FA superior corona radiata	non-APOE PGLS*Female	0.046	-0.107	0.199	0.5571
FA superior fronto.occipital fasciculus	non-APOE PGLS*Female	-0.063	-0.217	0.091	0.4234
FA superior longitudinal fasciculus	non-APOE PGLS*Female	-0.102	-0.256	0.051	0.1920

FA tapetum	non-APOE PGLS*Female	-0.042	-0.195	0.112	0.5947
FA uncinate fasciculus	non-APOE PGLS*Female	0.058	-0.094	0.211	0.4529

PGLS = polygenic longevity score, FA = fractional anisotropy, LCI = lower confidence limit, UCI = upper confidence limit, and P = p-value.

sTable 17: Secondary results - Sex-stratified associations between PGLSs and the regional cortical thicknesses

Group	Brain measures	PGLSs	Beta	LCI	UCI	P	Adjusted P
Males	Parsopercularis thickness	APOE PGLS	0.143	0.023	0.263	0.0197	**0.0328**
Males	Parstriangularis thickness	APOE PGLS	0.176	0.057	0.296	0.0040	**0.0161**
Males	Insula thickness	APOE PGLS	0.142	0.018	0.266	0.0246	**0.0328**
Males	Supramarginal thickness	APOE PGLS	0.084	-0.036	0.204	0.1706	0.1706
Females	Parsopercularis thickness	APOE PGLS	-0.039	-0.142	0.063	0.4501	0.6001
Females	Parstriangularis thickness	APOE PGLS	-0.017	-0.119	0.084	0.7411	0.7411
Females	Insula thickness	APOE PGLS	-0.065	-0.165	0.035	0.2039	0.6001
Females	Supramarginal thickness	APOE PGLS	-0.045	-0.146	0.056	0.3766	0.6001
Males	Parsopercularis thickness	non-APOE PGLS	0.141	0.022	0.261	0.0208	**0.0278**
Males	Parstriangularis thickness	non-APOE PGLS	0.192	0.073	0.311	0.0017	**0.0067**
Males	Insula thickness	non-APOE PGLS	0.146	0.022	0.269	0.0206	**0.0278**
Males	Supramarginal thickness	non-APOE PGLS	0.093	-0.027	0.212	0.1292	0.1292
Females	Parsopercularis thickness	non-APOE PGLS	-0.046	-0.147	0.056	0.3796	0.5061
Females	Parstriangularis thickness	non-APOE PGLS	-0.020	-0.121	0.081	0.6996	0.6996
Females	Insula thickness	non-APOE PGLS	-0.064	-0.163	0.035	0.2062	0.5061
Females	Supramarginal thickness	non-APOE PGLS	-0.056	-0.156	0.044	0.2735	0.5061

PGLS = polygenic longevity score, LCI = lower confidence limit, UCI = upper confidence limit, and P = p-value.

sTable 18: Secondary results - Sex-stratified associations between PGLSs and subcortical volume

Group	Brain measures	PGLSs	Beta	LCI	UCI	P	Adjusted P
Males	Pallidum volume	APOE PGLS	0.108	-0.017	0.233	0.0896	0.0896
Females	Pallidum volume	APOE PGLS	-0.039	-0.136	0.058	0.4258	0.4258
Males	Pallidum volume	non-APOE PGLS	0.111	-0.014	0.235	0.0808	0.0808
Females	Pallidum volume	non-APOE PGLS	-0.038	-0.135	0.058	0.4329	0.4329

PGLS = polygenic longevity score, LCI = lower confidence limit, UCI = upper confidence limit, and P = p-value.

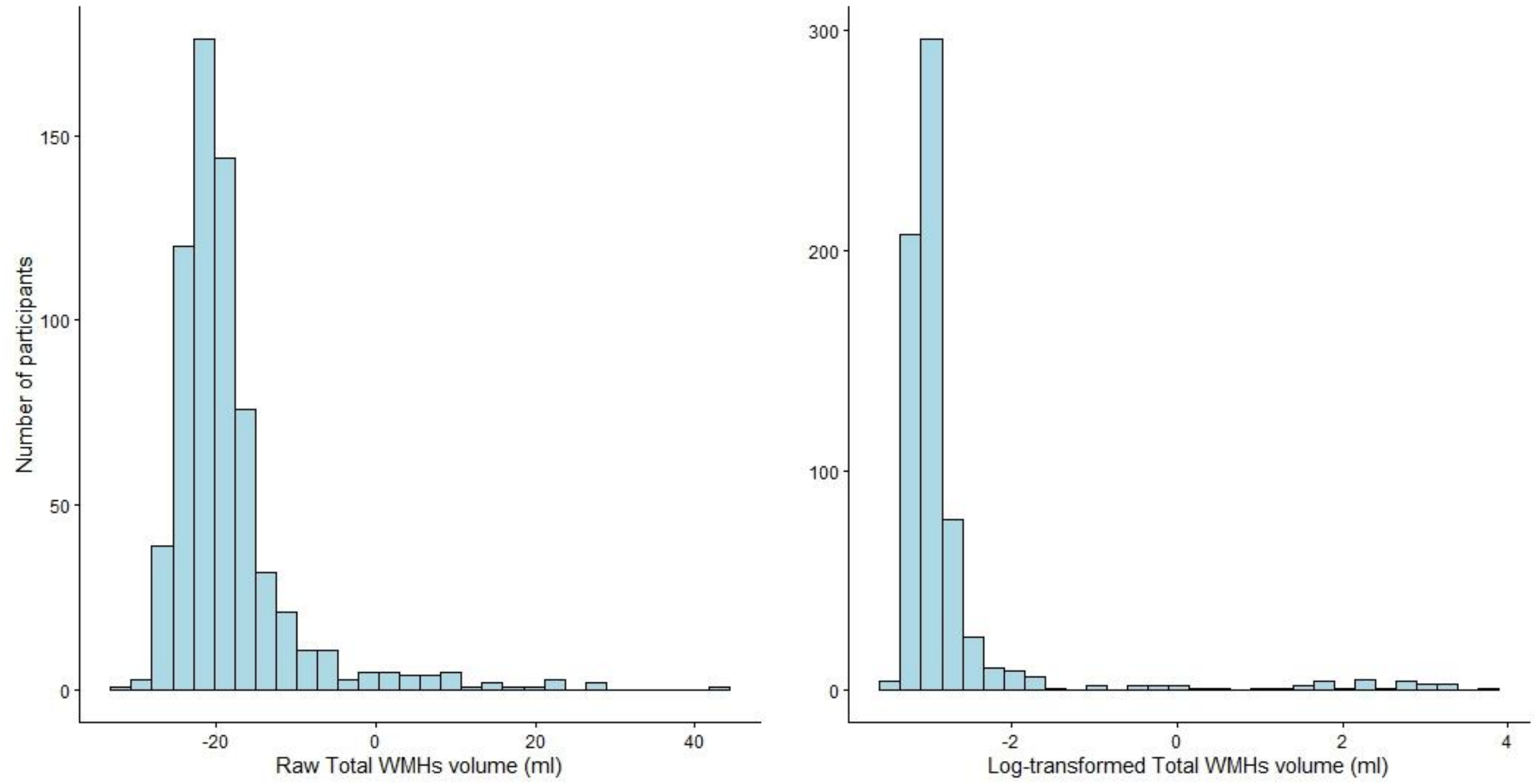

sFigure 1: Histogram - distribution of the raw total WMHs volume and the log-transformed total WMHs in the total sample (n = 671). ml = millilitres.

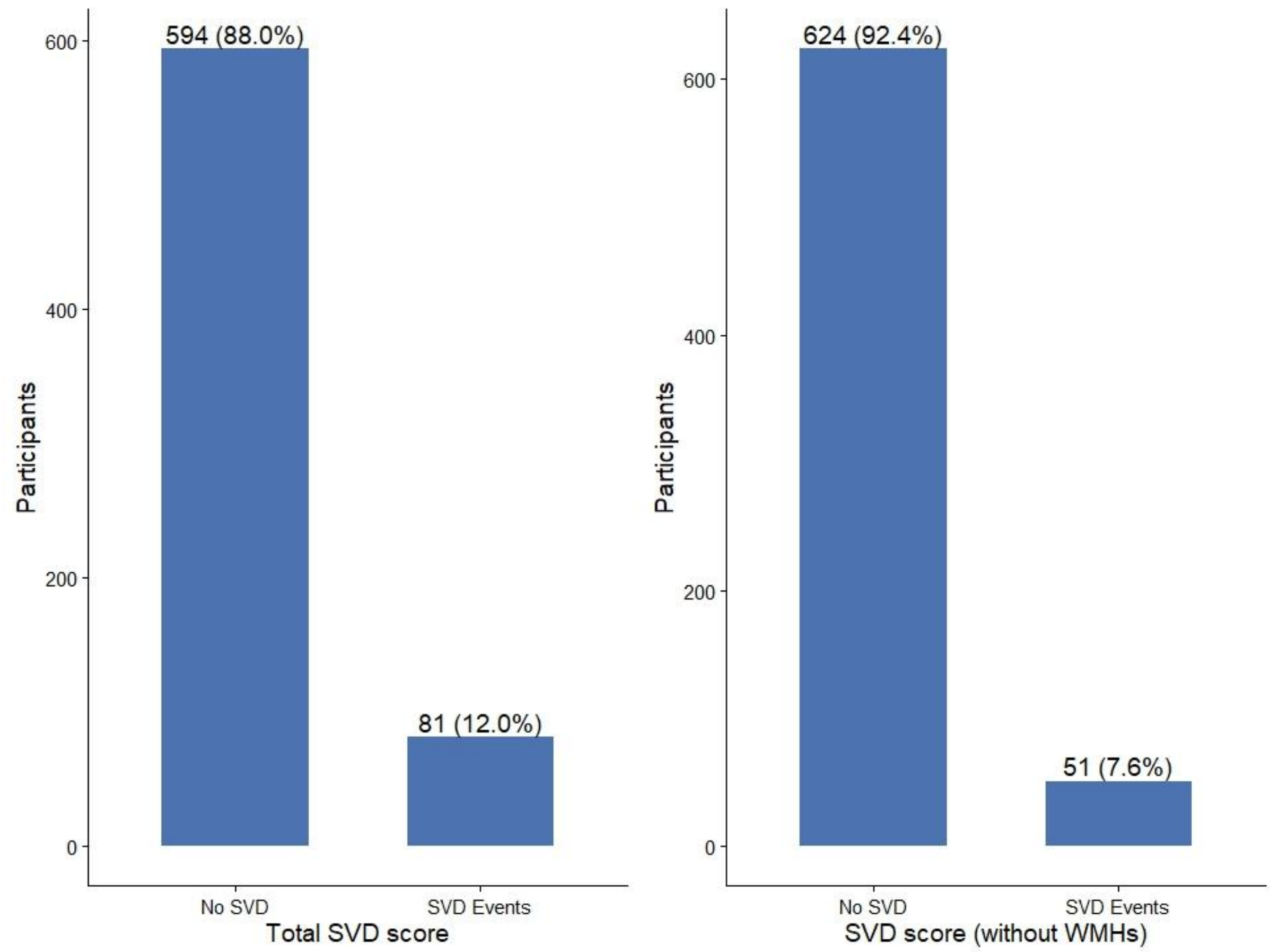

sFigure 2: Histogram - distribution of the binary SVD score and binary SVD score without WMHs in the total sample (n = 675).

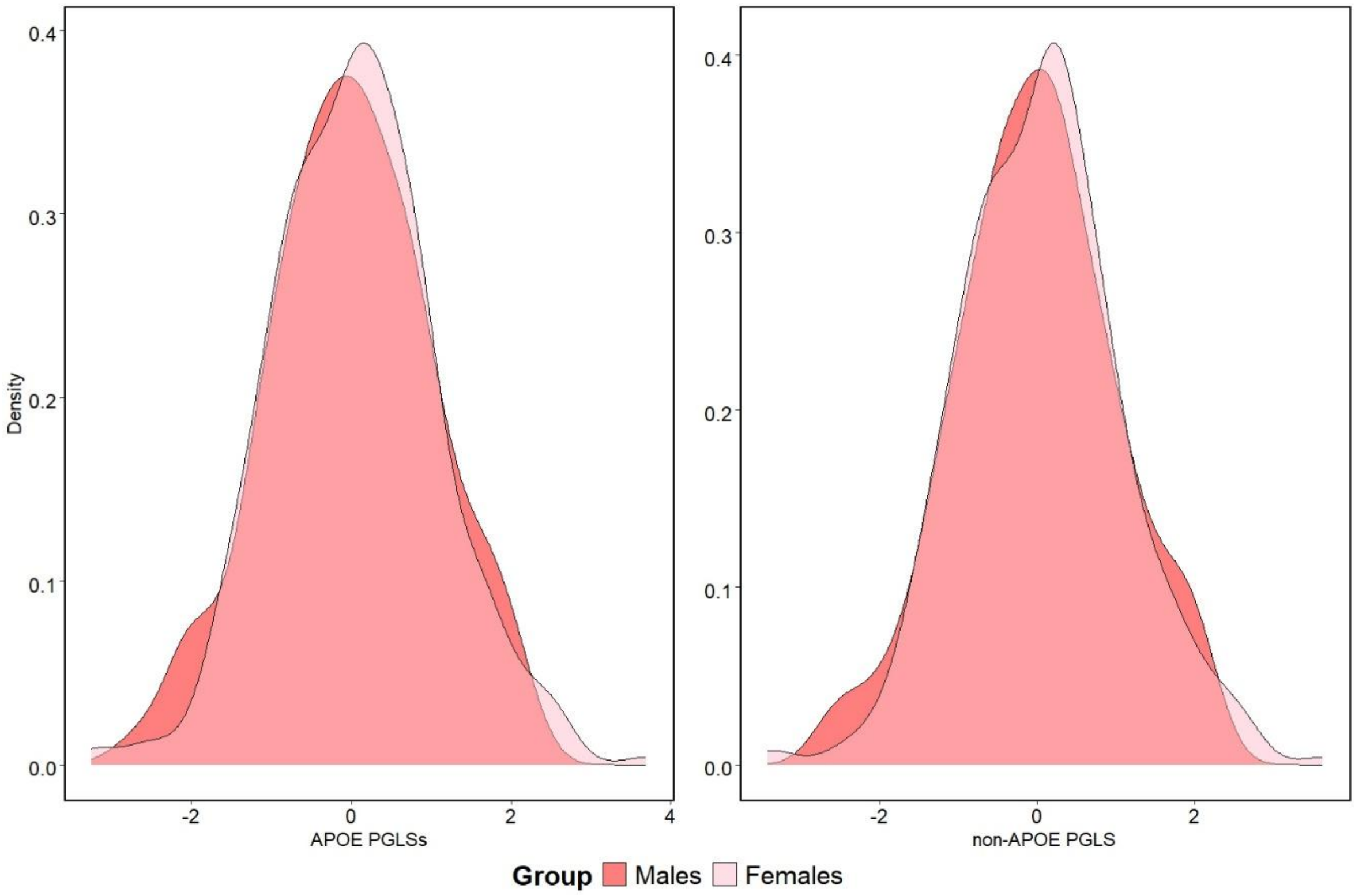

sFigure 3: Density plot - distribution of *APOE* and non-*APOE* PGLSs in males (n = 327) and females (n = 348).
